# Allosteric regulation of protein 14-3-3ζ scaffold by small-molecule editing modulates histone H3 post-translational modifications

**DOI:** 10.7150/thno.38483

**Published:** 2020-01-01

**Authors:** Yan-Jun Wan, Li-Xi Liao, Yang Liu, Heng Yang, Xiao-Min Song, Li-Chao Wang, Xiao-Wen Zhang, Yi Qian, Dan Liu, Xiao-Meng Shi, Li-Wen Han, Qing Xia, Ke-Chun Liu, Zhi-Yong Du, Yong Jiang, Ming-Bo Zhao, Ke-Wu Zeng, Peng-Fei Tu

**Affiliations:** 1 State Key Laboratory of Natural and Biomimetic Drugs, School of Pharmaceutical Sciences, Peking University, Beijing 100191, China.; 2 Proteomics Laboratory, Medical and Healthy Analytical Center, Peking University Health Science Center, Beijing 100191, China.; 3 Biology Institute, Qilu University of Technology (Shandong Academy of Sciences), Jinan, Shandong 250103, China.

**Keywords:** Histone post-translational modifications, 14-3-3ζ, allosteric effect, autophagy-lysosome function, endoplasmic reticulum stress

## Abstract

**Background:** Histone post-translational modifications (PTMs) are involved in various biological processes such as transcriptional activation, chromosome packaging, and DNA repair. Previous studies mainly focused on PTMs by directly targeting histone-modifying enzymes such as HDACs and HATs.

**Methods and Results:** In this study, we discovered a previously unexplored regulation mechanism for histone PTMs by targeting transcription regulation factor 14-3-3ζ. Mechanistic studies revealed 14-3-3ζ dimerization as a key prerequisite, which could be dynamically induced via an allosteric effect. The selective inhibition of 14-3-3ζ dimer interaction with histone H3 modulated histone H3 PTMs by exposing specific modification sites including acetylation, trimethylation, and phosphorylation, and reprogrammed gene transcription profiles for autophagy-lysosome function and endoplasmic reticulum stress.

**Conclusion:** Our findings demonstrate the feasibility of editing histone PTM patterns by targeting transcription regulation factor 14-3-3ζ, and provide a distinctive PTM editing strategy which differs from current histone modification approaches.

## Introduction

Histone post-translational modifications (PTMs) are major determinants that dictate the structure of chromatin and play a key role in regulating functional gene expression profiles 1-4. In mammals, histone PTMs, which exert versatile cellular functions, are dynamically regulated by multiple histone-modifying enzymes including histone deacetylases (HDACs) 5, 6, histone acetyltransferases (HATs) 7, histone demethylases (HDMs) 8, methylases 9 and phosphorylases 10, 11. Currently, studies mainly focus on the regulation of histone PTMs by directly targeting histone-modifying enzymes with small molecules such as histone deacetylase inhibitors (HDACIs) 12, 13. However, most of HDACIs bind to and chelate the zinc ion in the active site of HDACs, resulting in poor selectivity as well as off-target effects and potential toxicities *in vivo* 14. Therefore, there is an urgent need to explore novel druggable proteins for modulating histone PTMs.

As a family of crucial scaffold proteins, 14-3-3 proteins play a central role in a variety of physiological processes in mammals as well as other species 15. These biological functions are mainly achieved through 14-3-3-dependent protein-protein interactions (PPIs) as well as downstream cell signaling cascades 16. The dimeric nature of 14-3-3 is considered to be an important hallmark of 14-3-3 biology 17. Previous reports showed that 14-3-3 could recognize specific histone phosphorylation sites which regulate 14-3-3 PPI with histone. Moreover, 14-3-3 dimer has been reported to serve as a key scaffold protein platform for PPIs involving chromatin-modifying proteins with extensive effects on genetic expression and cell function 18, 19. However, the potential molecular mechanisms, as well as biological functions, are unexplored.

Here, we hypothesized that 14-3-3 might be a potential transcription regulation factor capable of targeting histones and regulating gene expressions by affecting histone PTM patterns. In this study, we identified a natural small-molecule protosappanin A (PTA) with a specific binding ability for 14-3-3 isoform ζ (14-3-3ζ). We found that PTA directly promoted 14-3-3ζ dimerization and specifically inhibited its interaction with histone H3, which resulted in the exposure of multiple histone H3 modification sites for modulating PTM patterns. Further, 14-3-3ζ-dependent histone H3 modifications exerted a neuroprotective effect by modifying endoplasmic reticulum (ER) stress and autophagy-lysosome gene expression profiles.

Our study identified 14-3-3ζ as a crucial factor involved in transcriptional regulation that can be targeted by small molecules. 14-3-3ζ dimerization can dynamically edit histone PTM patterns and provides a novel strategy for epigenetic regulation different from the current drugs that directly target histone-modifying enzymes.

## Results

### Identification of a small molecule targeting 14-3-3ζ

**Figure 1:**
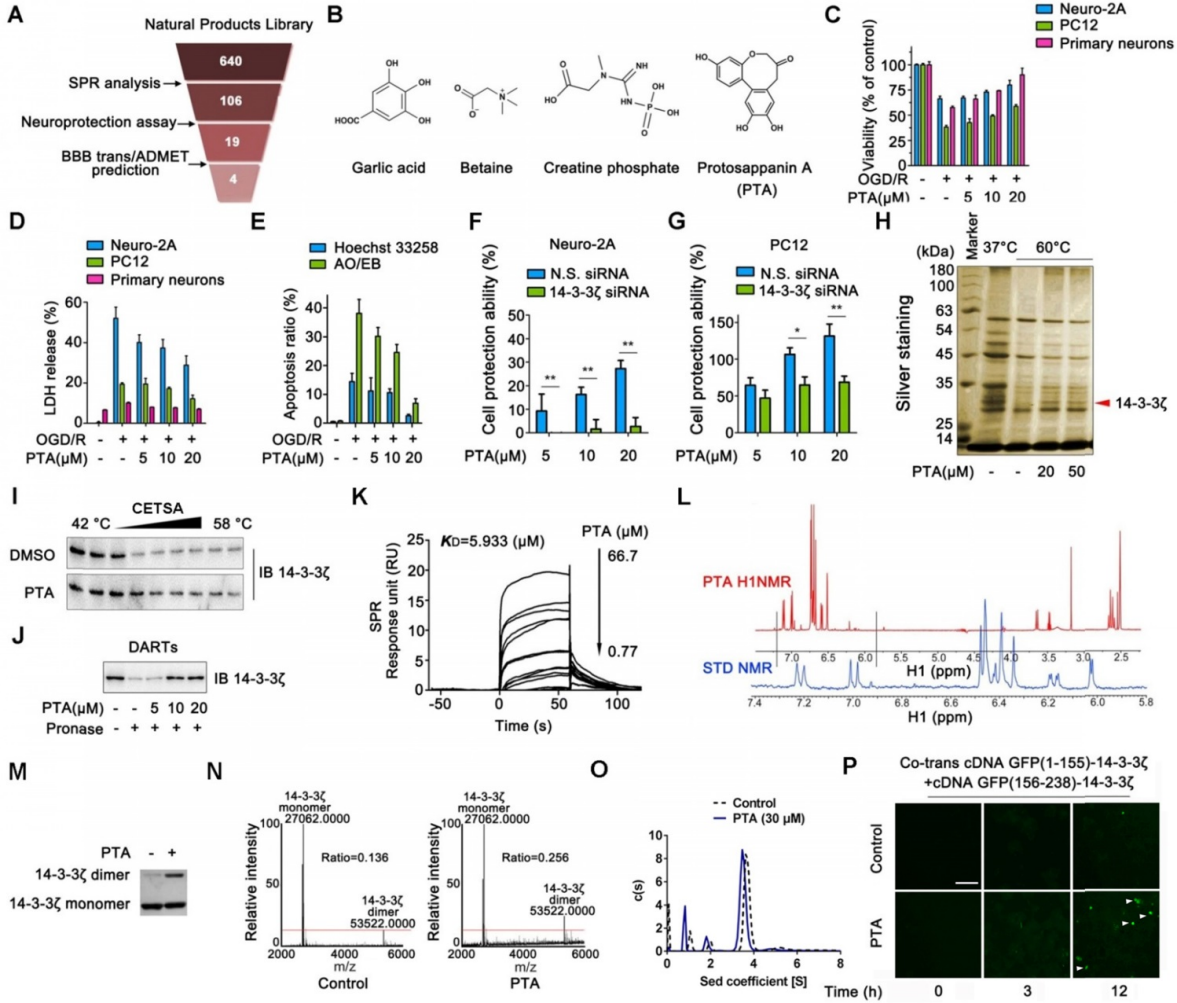
** Identification of a chemical small-molecule targeting 14-3-3ζ. (A)** Brief process of screening neuroprotective small-molecules targeting 14-3-3ζ. **(B)** Structures of garlic acid, betaine, creatine phosphate and protosappanin A (PTA). **(C)** PTA inhibited OGD/R-induced viability decrease in Neuro-2A cells, PC12 cells, and primary neurons. **(D)** PTA inhibited OGD/R-induced LDH release in Neuro-2A cells, PC12 cells, and primary neurons. **(E)** PTA inhibited OGD/R-induced apoptosis in Neuro-2A cells by Hoechst 33258 and AO/EB staining. **(F)** 14-3-3ζ siRNA reversed neuroprotective effect of PTA in Neuro-2A cells. **(G)** 14-3-3ζ siRNA reversed neuroprotective effect of PTA in PC12 cells. **(H)** 14-3-3ζ was identified as direct target of PTA using cellular thermal shift assay (CETSA) coupled with MS/MS analysis. **(I)** PTA promoted resistance of 14-3-3ζ to different temperature gradients (CETSA). **(J)** PTA promoted resistance of 14-3-3ζ to proteases (DARTs). **(K)** Direct interaction between PTA and 14-3-3ζ was confirmed by SPR analysis. **(L)** Direct interaction between PTA and 14-3-3ζ was confirmed by saturation transfer difference (STD)-NMR. **(M and N)** PTA promoted 14-3-3ζ dimerization *in vitro*, which was cross-linked by DSS and then evaluated by silver-staining SDS PAGE and LC-MS. **(O)** PTA promoted 14-3-3ζ dimerization *in vitro*, which was evaluated by analytical ultracentrifugation. **(P)** PTA promoted intracellular 14-3-3ζ dimerization in dynamics, which was evaluated by bimolecular fluorescence complementation (BiFC) assay.

We established a screening system to identify candidate compounds specifically targeting 14-3-3ζ (Fig. 1A). First, we performed surface plasmon resonance (SPR) analysis to identify 106 potential compounds binding to 14-3-3ζ from our previously established Natural Products Library (total of 640 compounds) (Fig. S1A). These compounds were then tested in a neuronal injury screening model because 14-3-3ζ is a valuable therapeutic target for neuroprotection (Fig. S1B). The compounds were also evaluated for their potential toxicities as well as an *in silico* evaluation for their ability to cross the blood-brain barrier (BBB) 20 (Fig. S1C). Finally, four candidates were identified including garlic acid, betaine, creatine phosphate, and protosappanin A (Fig. 1B). Because of its molecular structure complexity, protosappanin A (PTA), a natural dibenzoxocin derivative, was ultimately selected as the candidate small molecule (Fig. S1D-F). Subsequently, the neuroprotective effect of PTA was confirmed on three cell types: Neuro-2A cells, PC12 cells, and primary neurons (Fig. 1C-1E and S1G-I), which have been widely used as canonical model systems to study neuropharmacological molecular basis 21-26, and the potential role of 14-3-3ζ in PTA-mediated neuroprotection was investigated. As shown in Fig. 1F-G and S1J, the neuroprotective effect of PTA was significantly reversed in 14-3-3ζ-knockdown cells, indicating that 14-3-3ζ plays a pivotal role in the neuroprotective effect of PTA. Furthermore, PTA showed noticeable neuroprotective effects in the *in vivo* zebrafish swimming behavioral injury model (Fig. S1K-N) and the rat middle cerebral artery occlusion (MCAO) model (Fig. S1O-P).

### Small-molecule PTA can dynamically control 14-3-3ζ dimerization

To confirm the direct targets of PTA in cells, we performed the cellular thermal shift assay (CETSA), which is based on the concept that the binding of a ligand to its protein target promotes the target protein stabilization at a specific permissive temperature 27. SDS-PAGE analysis showed that one band (marked by the arrow) completely disappeared in the cells treated with DMSO at 60 °C; however, the band persisted in the cells treated with PTA. This band was identified as 14-3-3ζ protein by LC-MS/MS analysis (Fig. 1H and S2A). The results revealed that PTA significantly increased the stability of 14-3-3ζ in cells (Fig. 1I). Subsequently, we conducted a drug affinity responsive target stability (DARTS) assay to explore drug target engagement 28. We found that PTA significantly inhibited pronase-induced degradation of 14-3-3ζ, supporting the direct interaction of PTA with 14-3-3ζ (Fig. 1J). We also performed SPR analysis to quantify the interaction of PTA with 14-3-3ζ. As shown in Fig. 1K, PTA specifically bound to 14-3-3ζ with a dissociation constant (*K*_D_) of 5.933 μM, indicating a strong binding between PTA and 14-3-3ζ. Additionally, nuclear magnetic resonance (NMR) analysis revealed positive saturation transfer difference (STD) signals in the STD spectrum, confirming that 14-3-3ζ directly interacted with PTA (Fig. 1L). Furthermore, we examined the interaction of PTA and other proteins (such as 14-3-3α/β, 14-3-3ε, 14-3-3γ, 14-3-3η, and 14-3-3τ) of 14-3-3 family by CETSA assays. The results showed that PTA had no obvious effect on heat-induced degradation of 14-3-3α/β, 14-3-3ε, 14-3-3γ, 14-3-3η, and 14-3-3τ (Fig. S2B).

14-3-3ζ dimerization is essential for its stability and biological function in the context of various human diseases 29. We, therefore, attempted to determine whether PTA could regulate 14-3-3ζ dimerization. We found that PTA could markedly promote 14-3-3ζ dimerization *in vitro* (Fig. 1M and S2C), as determined quantitatively by LC-MS (Fig. 1N). Isothermal titration calorimetry (ITC) analysis also revealed heat release about 20 min after 14-3-3ζ dimerization (Fig. S2D). Simultaneously, sedimentation equilibrium analysis showed that PTA promoted 14-3-3ζ dimerization (from 69.134% to 77.457%) (Fig. 1O). Moreover, we established a bimolecular fluorescence complementation (BiFC) reporter system for real-time imaging of 14-3-3ζ dimer formation in cells. As shown in Fig. 1P, PTA induced a slight increase in green fluorescent protein (GFP) signal from 3 h and a marked GFP increase at 12 h, indicating that PTA could dynamically promote 14-3-3ζ dimerization in cells.

### Cysteine189 is a druggable allosteric site for 14-3-3ζ dimerization

**Figure 2:**
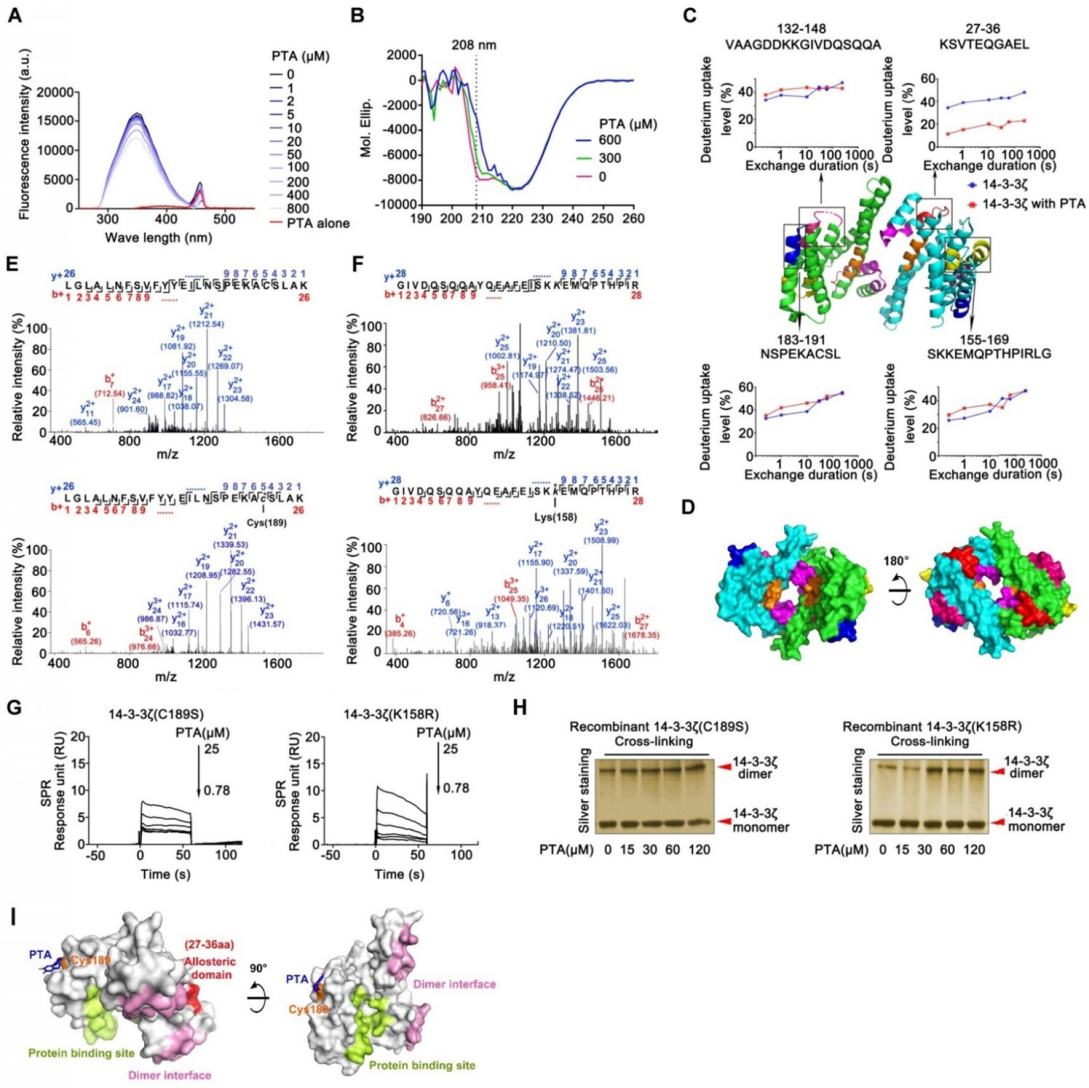
** Cysteine189 is a druggable allosteric site for 14-3-3ζ dimerization. (A)** Fluorescence spectroscopy analysis of interaction of 14-3-3ζ with PTA. **(B)** CD spectra analysis for PTA-mediated 14-3-3ζ conformational change. **(C)** HDX profiles of 14-3-3ζ. Peptides with different HDX profiles in 14-3-3ζ are highlighted in different colors on X-ray crystal structure of 14-3-3ζ (PDB: 2C1N). Deuterium uptake plots of these peptides are presented. **(D)** Allosteric domains were shown in 3D structure of 14-3-3ζ dimer. **(E)** LC-MS/MS analysis of PTA-binding site on C189 of 14-3-3ζ. **(F)** LC-MS/MS analysis of PTA-binding site on K158 of 14-3-3ζ. **(G)** SPR analysis of PTA binding to 14-3-3ζ mutant (C189S) and SPR analysis of PTA binding to 14-3-3ζ mutant (K158R). **(I)** C189S but not K158R mutation suppressed PTA-dependent 14-3-3ζ dimerization. Silver-staining SDS PAGE analysis of PTA-dependent 14-3-3ζ mutant (C189S and K158R) dimerization. **(K)** Molecular docking of PTA binding to 14-3-3ζ.

The allosteric effect is an important strategy for regulating protein function that requires the participation of small molecules 30. We performed fluorescence analysis of tryptophan in 14-3-3ζ to investigate the potential role of PTA in the regulation of 14-3-3ζ conformational changes. As displayed in Fig. 2A, the fluorescence intensity of PTA-14-3-3ζ complex decreased upon PTA treatment, showing that PTA could induce conformational changes in 14-3-3ζ, as confirmed by CD spectra analysis (Fig. 2B). The decrease in alpha-helix signal, which was induced by PTA, might have resulted from forming random coil structure and collapse of the alpha-helix structure. To understand the allosteric mechanism, we compared the hydrogen/deuterium exchange mass spectrometry (HDX-MS) profiles of 14-3-3ζ alone and with PTA. 14-3-3ζ comprises a single domain of nine α-helices (α1-α9) with short loops between them. The HDX-MS profile of 14-3-3ζ was consistent with the high-resolution structure of 14-3-3ζ (PDB: 2C1N) 31. The peptides at loops exposed to the buffer had higher deuterium uptake than the regions with ordered α-helical secondary structure and buried depth (Fig. S2E). These observations suggested that our experimental system was functional and that 14-3-3ζ protein was well folded. The results of HDX-MS also suggested conformational changes in 14-3-3ζ associated with PTA. Four peptides were identified by LC-MS/MS, and the deuterium uptake profiles of these peptides were analyzed. PTA treatment altered the hydrogen/deuterium exchange levels of four specific peptides (peptides 27-36, 132-148, 155-169, and 183-191) (Fig. 2C and S2F). Peptide 27-36, located at α2 and α3, was changed most even during a short exchange-time duration, suggesting this region may serve as a critical functional domain for PTA-mediated 14-3-3ζ conformational changes. Because the 14-3-3ζ dimer interface is formed by packing the α1 helix from one 14-3-3ζ monomer against the α3 and α4 helices of the other, we speculated that PTA promotes 14-3-3ζ dimerization by allosterically regulating the dimer interface (Fig. 2D).

LC-MS/MS analysis of trypsin-digested reaction products identified a peptide (LGLALNFSVFYYEILNSPEKACSLAK) with the calculated mass of 2677.06 Da, which is 253.98 Da larger than the corresponding peptide in recombinant 14-3-3ζ (Fig. 2E). The mass difference of 253.98 Da matches the molecular weight of PTA (losing one H_2_O molecule), suggesting that PTA specifically modified the LGLALNFSVFYYEILNSPEKACSLAK peptide in 14-3-3ζ. Furthermore, MS/MS spectrometry of this peptide revealed a 253.98 Da mass shift (starting from fragment ions y^5^), indicating that the cysteine in this peptide (corresponding to C189 in 14-3-3ζ) was covalently modified by PTA (Fig. 2E and S2G). We also identified another peptide (GIVDQSQQAYQEAFEISKKEMQPTHPIR) in 14-3-3ζ that was specifically modified by PTA on Lys158 (K158) (Fig. 2F). Mutations at these two sites (C189S, K158R) effectively abolished the bindings between PTA and 14-3-3ζ mutants, as confirmed by SPR analysis (Fig. 2G and Fig. S2H). Notably, we found that incubation of 14-3-3ζ mutant (K158R) with PTA *in vitro* still promoted its dimerization; but this was not the case for 14-3-3ζ mutant (C189S) (Fig. 2H), suggesting C189 as a key binding site for PTA on 14-3-3ζ responsible for its dimerization. These results were confirmed by molecular docking assay (Fig. 2I and S2I-J).

### 14-3-3ζ dimer specifically interacts with histone H3 in the nucleus

**Figure 3:**
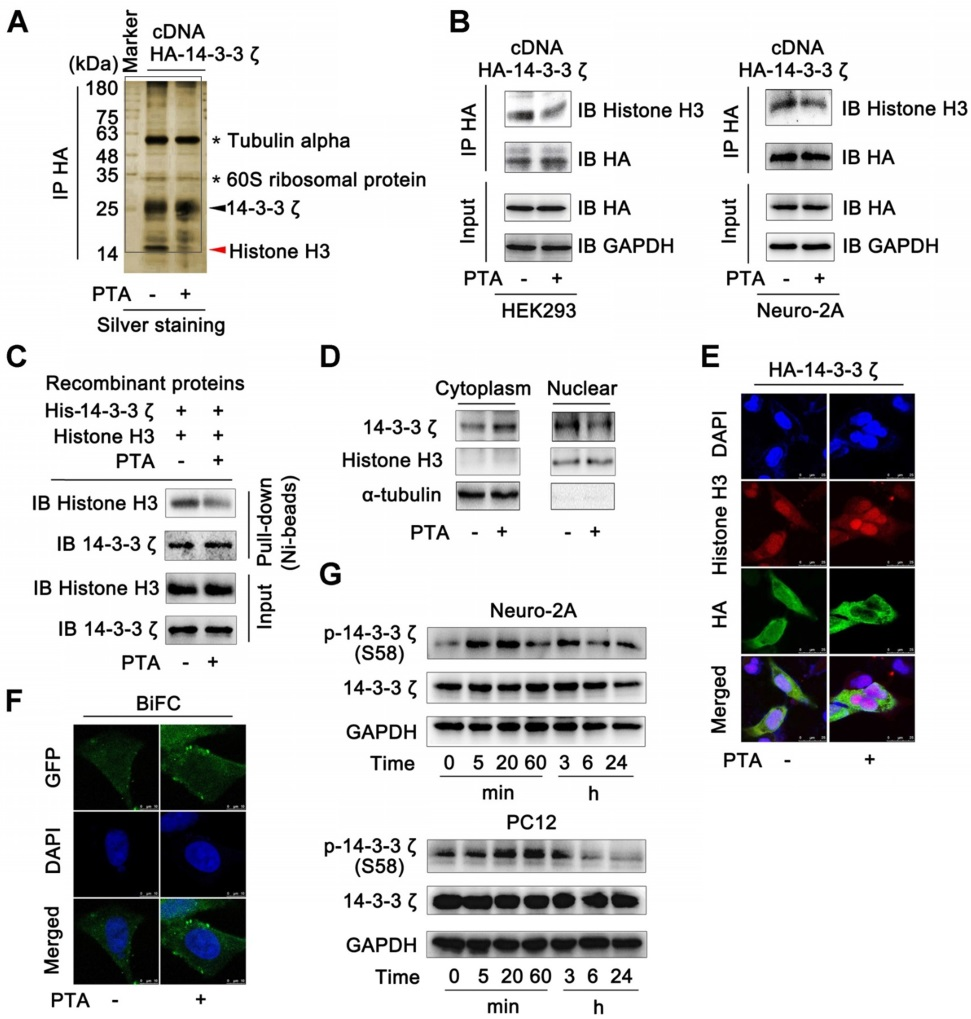
** 14-3-3ζ dimer specifically interacts with histone H3 in nucleus. (A)** 14-3-3ζ interaction with histone H3 was specially inhibited by PTA. Co-IP was performed with anti-HA antibody followed by silver staining and LC-MS/MS analysis for specific protein bands. HEK293T cells transfected with HA-tagged 14-3-3ζ vector were treated with 20 μM PTA or vehicle. **(B)** 14-3-3ζ interaction with histone H3 was inhibited by PTA in HA-tagged 14-3-3ζ-transfected HEK293T and Neuro-2A cells**. (C)** Recombinant 14-3-3ζ protein interaction with recombinant histone H3 protein was inhibited by PTA. **(D and E)** Cytoplasmic translocation of 14-3-3ζ from nucleus was promoted by PTA. HA-tagged 14-3-3ζ was shown in green, and histone H3 was shown in red (scale bars = 25 μm). **(F)** 14-3-3ζ dimerization was promoted by PTA in cytoplasm (BiFC, scale bars = 10 μm). **(G)** Phosphorylation levels of 14-3-3ζ (S58) were increased by PTA.

Several proteins are known to bind to 14-3-3ζ 32. We, therefore, performed co-immunoprecipitation (Co-IP) to identify the potential 14-3-3ζ-binding proteins. The results of SDS-PAGE identified many 14-3-3ζ-binding proteins including histones, tubulin α, and 60S ribosomal proteins; however, only one band (marked by the red arrow) disappeared upon treatment with PTA (Fig. 3A). This protein was further identified as histone H3 by LC-MS/MS analysis (Fig. S3A). We also tested the interactions of 14-3-3ζ with the previously reported 14-3-3ζ partner proteins such as FoxO3, p53, Tau, and Bad. PTA did not affect the binding of 14-3-3ζ to these proteins (Fig. S3B). Together, these data suggested that PTA could selectively inhibit the interaction of 14-3-3ζ with histone H3 but no other client proteins.

Next, we sought to confirm these observations in HEK293T and Neuro-2A cells that overexpressed 14-3-3ζ (Fig. S3C). The results showed that 14-3-3ζ directly interacted with histone H3 (Fig. S3D and E), but this interaction was effectively inhibited by PTA (Fig. 3B and C). 14-3-3ζ mutant (C189S) was resistant to PTA-mediated 14-3-3ζ-histone H3 interactions, suggesting C189 as a pivotal target site for PTA on 14-3-3ζ (Fig. S3F). Furthermore, PTA induced cytoplasmic translocation of 14-3-3ζ from the nucleus (Fig. 3D), which was also supported by immunofluorescence analysis (Fig. 3E). BiFC assay showed that PTA promoted more 14-3-3ζ dimer formation in the cytoplasm (Fig. 3F) perhaps reflecting a PTA-dependent allosteric effect on 14-3-3ζ protein. Additionally, PTA induced 14-3-3ζ phosphorylation (S58) within 5-20 min (Fig. 3G). This finding suggested that phosphorylation may be a precondition for 14-3-3ζ dimerization. 33 14-3-3ζ mutant (S58A) blocked PTA-dependent cytoplasmic translocation of 14-3-3ζ from the nucleus (Fig. S3G), which was also confirmed by immunofluorescence analysis (Fig. S3H). Furthermore, 14-3-3ζ mutant (S58A) inhibited the PTA-dependent interaction between 14-3-3ζ and histone H3 (Fig. S3I and S3J). These observations indicated that the binding between 14-3-3ζ and histone H3 might be regulated by PTA by targeting C189 allosteric site in 14-3-3ζ.

### 14-3-3ζ dimer regulates histone H3 PTM pattern

**Figure 4:**
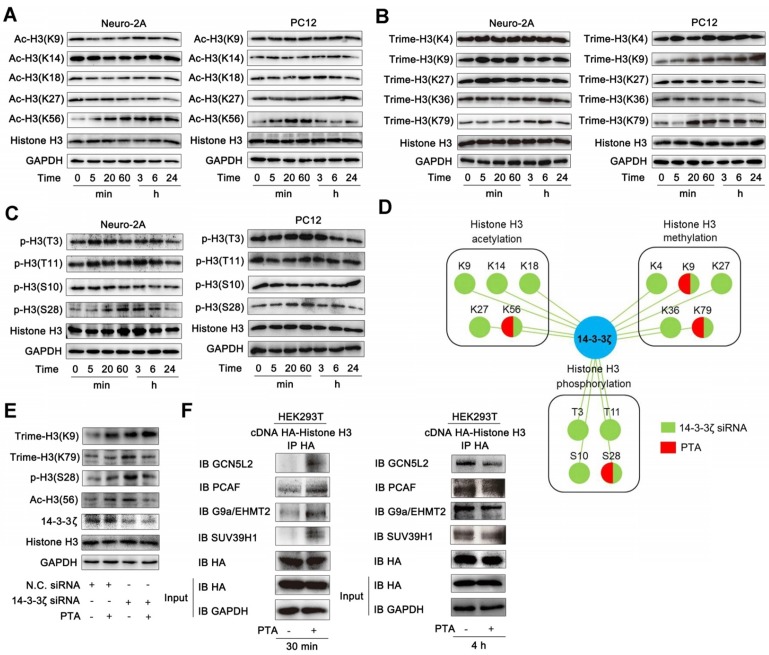
** 14-3-3ζ dimer regulates histone H3 PTMs pattern. (A)** PTA affected acetyl-histone H3 (Lys56) in Neuro-2A and PC12 cells. **(B)** PTA affected trimethyl-histone H3 (Lys9 and 79) in Neuro-2A and PC12 cells. **(C)** PTA affected phos-histone H3 (Ser28) in Neuro-2A and PC12 cells. **(D)** Pattern diagram for 14-3-3ζ-mediated post-translational modifications on histone H3 by genetic knockdown and chemical regulation. **(E)** 14-3-3ζ knockdown blocked PTA-dependent post-translational modifications of histone H3. **(F)** PTA promoted interactions of histone acetyltransferases (HATs) and histone methyltransferases with histone H3.

Since PTA regulates the interaction between 14-3-3ζ and histone H3, we hypothesized that PTA might influence the PTMs of histone H3. To this end, we detected the acetylation, trimethylation, and phosphorylation of histone H3. As shown in Fig. 4A, PTA markedly increased histone H3 acetylation at Lys56 (K56). PTA also increased the trimethylation of histone H3 at Lys9 (K9) and Lys79 (K79) (Fig. 4B). Moreover, PTA increased the phosphorylation of histone H3 at Ser28 (S28) (Fig. 4C). To further investigate the effects of 14-3-3ζ on histone H3 PTMs, we decreased 14-3-3ζ expression with specific siRNA. Compared with PTA treatment, 14-3-3ζ knockdown induced a much broader increase in PTM patterns of histone H3 (Fig. 4D and S4A). To confirm that the PTMs (K9, K56, K79, S28) by PTA were mediated through targeting 14-3-3ζ, we measured the extent of these modifications in the presence of 14-3-3ζ siRNA or negative control siRNA. As shown in Fig. 4E, PTA-mediated modifications were completely blocked in cells transfected with 14-3-3ζ siRNA. Co-IP analysis revealed that PTA promoted the binding of histone H3 (over a relatively brief duration) to histone acetyltransferases such as GCN5L2 and PCAF, as well as histone methyltransferases such as G9a/EHMT2 and SUV39H1 (Fig. 4F). However, PTA-induced PPIs returned to normal levels after 4 h (Fig. 4F). This trend was consistent with the effect of 14-3-3ζ siRNA (Fig. S4B). Moreover, we performed additional experiments for histone H3 PTMs in OGD/R model with or without PTA treatment and found that PTA could still regulate the acetylation, trimethylation, and phosphorylation levels of 14-3-3ζ, indicating that histone H3 PTMs were a key biological process for PTA-mediated neuroprotective effects (Fig. S4C).

We speculated that the acetylation and trimethylation modifications of histone H3 resulted from PTA-mediated binding of acetyltransferases and methyltransferases to histone H3. We, therefore, engineered single-point mutations at these modification sites on histone H3 and detected its interaction with 14-3-3ζ. The results showed that mutations at these modification sites increased 14-3-3ζ binding to histone H3 (Fig. S4D), suggesting that histone H3 modifications might prevent 14-3-3ζ from binding to histone H3. Additional experiments will be necessary to confirm these findings.

### 14-3-3ζ is a transcriptional regulation factor for diverse gene expressions

**Figure 5:**
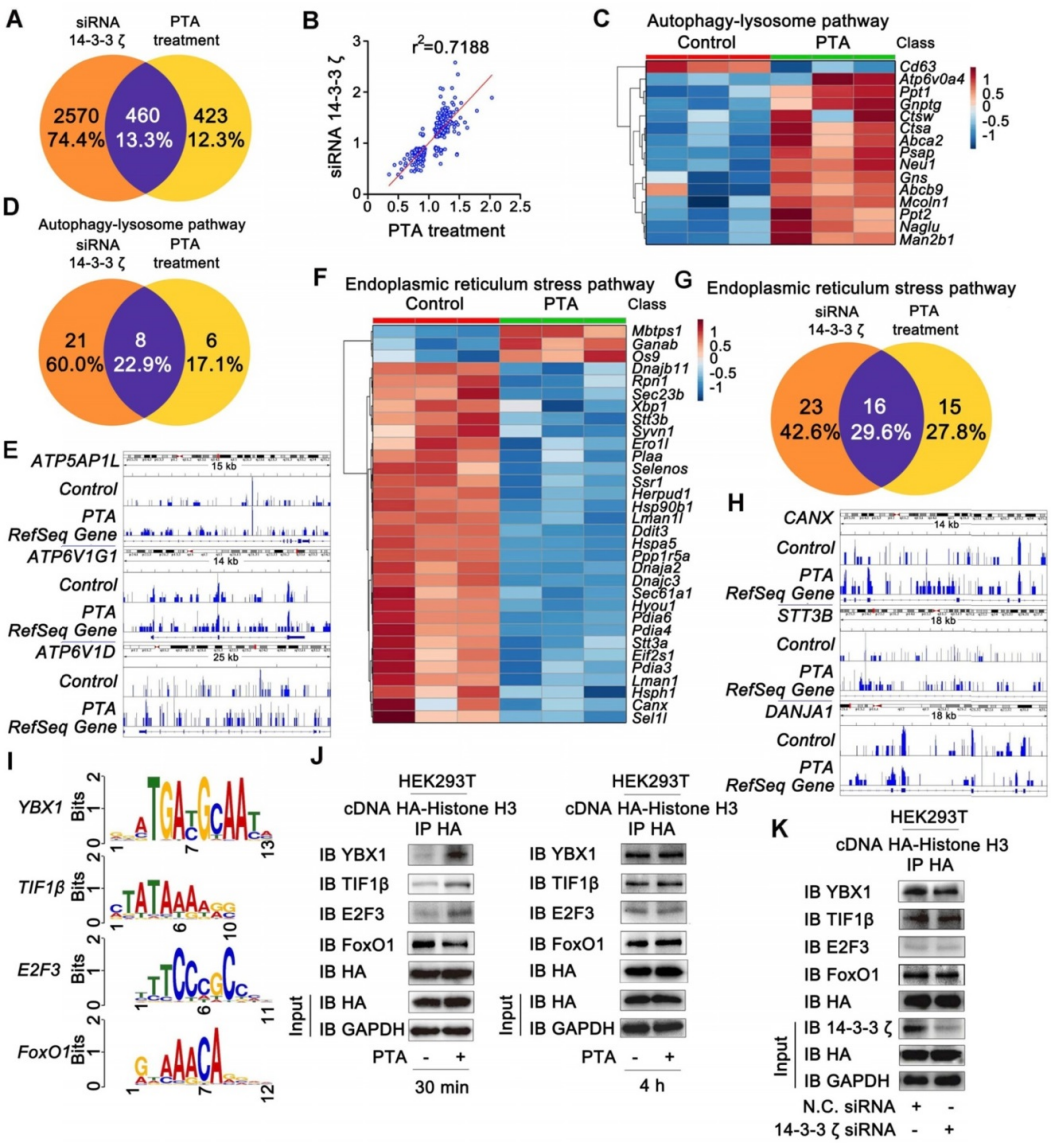
** 14-3-3ζ is a transcription regulation factor for diverse gene expressions. (A)** Similarity analysis of whole transcriptional changes after treatment with 14-3-3ζ siRNA or PTA at 20 μM. **(B)** Scatter-plots showing correlation with transcriptional changes after treatment with 14-3-3ζ siRNA or PTA at 20 μM. **(C)** Heat map of gene expression changes to core autophagy-lysosome pathway after treatment with PTA. **(D)** Similarity analysis of autophagy-lysosome pathway-related transcriptional changes after treatment with 14-3-3ζ siRNA or PTA at 20 μM. **(E)** IGV screenshots for *ATP5AP1L*, *ATP6V1G1* and *ATP6V1D* loci. **(F)** Heat map of gene expression changes to core endoplasmic reticulum stress pathway after treatment with PTA. **(G)** Similarity analysis of endoplasmic reticulum stress pathway-related transcriptional changes after treatment with 14-3-3ζ siRNA or PTA at 20 μM. **(H)** IGV screenshots for *CANX*, *STT3B* and *DANJA1* loci. **(I)** Conserved motifs of transcription factors* YBX1*, *TIF1β*, *FoxO1* and *E2F3* were shown by TomTom analysis. **(J)** Histone H3 interaction with transcription factors was promoted by PTA in HA-tagged histone H3-transfected HEK293T cells. **(K)** 14-3-3ζ knockdown did not affect histone H3 binding to transcription factors.

To characterize gene expressions regulated by 14-3-3ζ, we genetically knocked down 14-3-3ζ with siRNA and profiled the mRNA transcriptions followed by bioinformatics analysis. As shown in Fig. S5A and B, genetic transcriptions in the autophagy-lysosome pathway were significantly increased, and those in endoplasmic reticulum stress pathway were markedly suppressed in 14-3-3ζ siRNA-transfected cells, indicating that 14-3-3ζ may play a role in these two biological processes. Subsequently, we investigated pharmacologically-induced gene expression profile by PTA. Altered expression of about 460 genes in PTA-treated cells overlapped and highly correlated with the those in 14-3-3ζ-knockdown cells further supporting 14-3-3ζ as a functional target of PTA (Fig. 5A and B). We also investigated gene expressions in the autophagy-lysosome pathway and found that 14 genes were significantly up-regulated by PTA (Fig. 5C) including 8 genes with similar upregulation pattern in siRNA-transfected cells (Fig. 5D and S5C). CHIP-seq profile revealed that 14-3-3ζ accumulated at several autophagy-lysosome-associated genes, such as* ATP5AP1L*, *ATP6V1G1,* and *ATP6V1D* loci (Fig. 5E and S5D). Therefore, these results suggested that PTA could activate the autophagy-lysosome pathway through mediating 14-3-3ζ-dependent gene transcription to exert the neuroprotective effect. Additionally, we also studied the gene expressions in endoplasmic reticulum stress pathway and observed decreased expression of 29 genes by PTA (Fig. 5F) more than half of which were also downregulated in siRNA-transfected cells (Fig. 5G and S5E). CHIP-seq profile revealed that 14-3-3ζ accumulated at several ER stress-associated genes such as* CANX*, *STT3B,* and *DANJA1* loci (Fig. 5H and S5F). These observations indicated that 14-3-3ζ might act as a regulator of ER stress gene expressions, ultimately resulting in a neuroprotective effect.

The extensive regulation of expression of genes by PTA suggested that it might mediate the biological functions of specific transcription factors that affect genome-wide transcription profiles. To this end, we found that the genes with transcription factor binding motifs in their promoters including *YBX1*, *TIF1β*, *FoxO1,* and *E2F3* were particularly sensitive to PTA treatment and had significantly altered expression (Fig. 5I and S5G). We performed Co-IP to explore whether the binding of these transcription factors with histone H3 was influenced by PTA. As shown in Fig. S5H, several potential histone H3-binding protein bands (marked by the red arrow) were slightly increased upon treatment with PTA. These proteins were identified as Y box binding protein (YBX) 1 (Fig. S5I) and transcription intermediary factor (TIF) 1β (Fig. S5J) by LC-MS/MS analysis. Subsequently, we confirmed this observation in HEK293T cells that overexpressed histone H3 (Fig. 5J). These PTA-mediated interactions returned to normal levels after 4 h (Fig. 5J) which was consistent with the effect achieved using 14-3-3ζ siRNA (Fig. 5K). These data revealed that 14-3-3ζ could regulate the interactions of transcription factors with histone H3 to modulate various gene expression profiles for the neuroprotective effect.

### PTA activates expression of mitophagy genes for neuronal survival by targeting 14-3-3ζ

**Figure 6:**
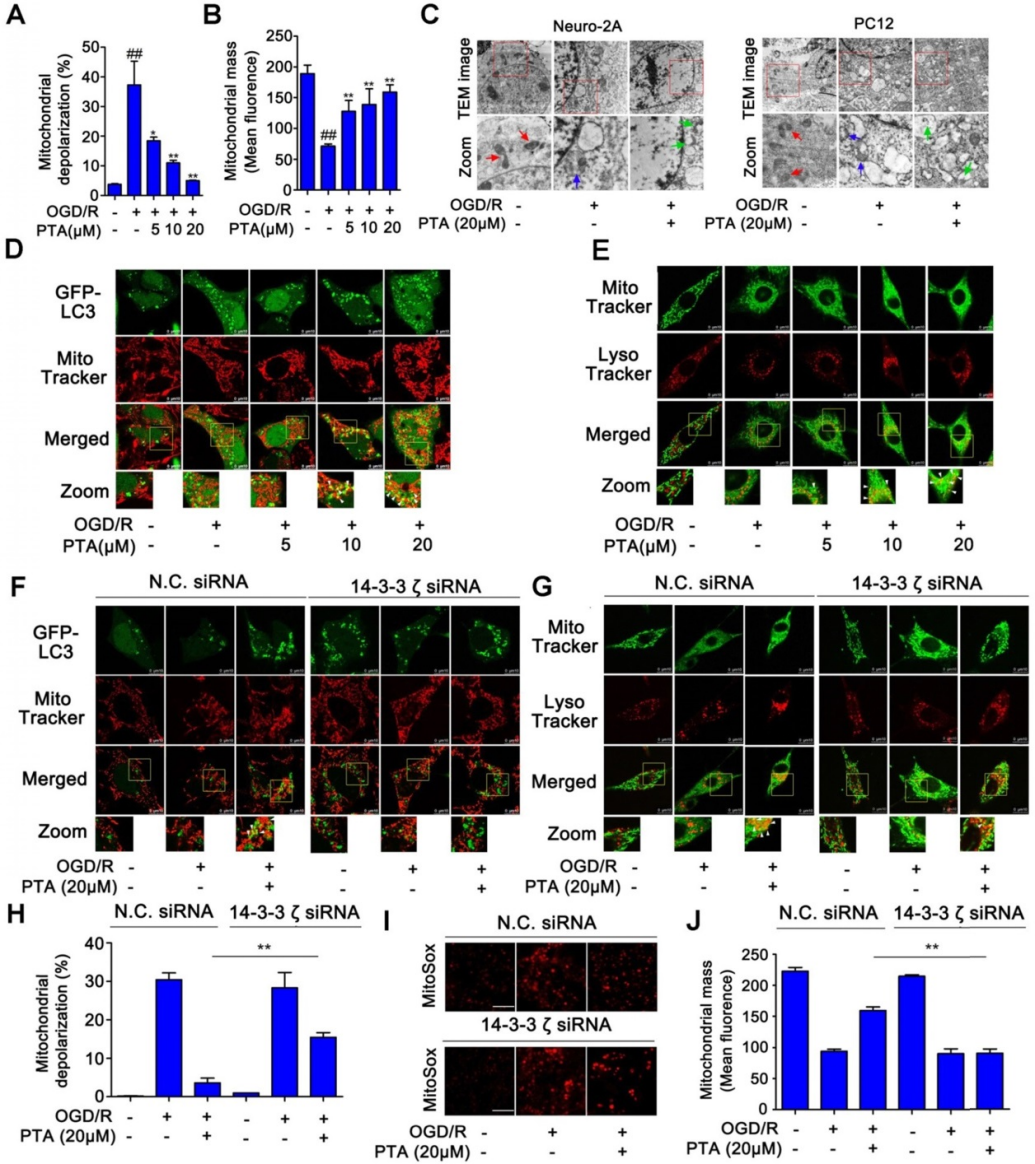
** 14-3-3ζ activates mitophagy gene expressions for neuronal survival. (A)** Mitochondrial depolarization was inhibited by PTA using JC-1 staining assay. **(B)** Loss of mitochondrial mass was inhibited by PTA using MitoTracker Red FM staining assay. **(C)** Transmission electron microscope (TEM) analysis of mitophagy in Neuro-2A cells and PC12 cells. **(D)** PTA promoted colocalization of GFP-tagged LC3B (green) with MitoTracker (red). **(E)** PTA promoted colocalization of LysoTracker (red) with MitoTracker (green). **(F)** PTA-induced colocalization of GFP-tagged LC3B (green) with MitoTracker (red) was blocked in 14-3-3ζ siRNA-transfected HEK293T cells. **(G)** PTA-induced colocalization of LysoTracker (red) with MitoTracker (green) was blocked in 14-3-3ζ siRNA-transfected Neuro-2A cells. **(H)** PTA-induced inhibition of mitochondrial depolarization was blocked in 14-3-3ζ siRNA-transfected Neuro-2A cells. **(I)** PTA-induced inhibition of mitochondrial mass loss was blocked in 14-3-3ζ siRNA-transfected Neuro-2A cells. **(J)** PTA-induced inhibition of mitochondrial superoxide production was blocked in 14-3-3ζ siRNA-transfected Neuro-2A cells. Scale bars = 10 μm.

We then asked if there was a correlation between PTA-dependent autophagy-lysosome gene expression and neuroprotection. To answer this question, we examined the effect of PTA on mitophagy, which is a key protective mechanism that specifically eliminates dysfunctional mitochondria. PTA effectively protected mitochondria against OGD/R-induced injury by maintaining mitochondrial membrane potential and increasing mitochondrial mass (Fig. 6A, B, and S6A), and 14-3-3ζ siRNA blocked PTA-mediated mitochondrial protection (Fig. S6B). To monitor the pH of the lysosomes, we used LysoSensorTM Yellow/Blue fluorescent probe as a lysosomal pH indicator. As shown in Fig. S6C, the pH decreased in OGD/R-induced cells, which was effectively inhibited by PTA. Specific 14-3-3ζ knockdown significantly impaired PTA-mediated pH recovery (Fig. S6D). Also, TEM was used to observe mitophagy and ultrastructural changes. As shown in Fig. 6C, swelling of damaged mitochondria was visible in OGD/R-induced cells, and swollen mitochondria were found in close proximity to autophagy vacuoles/lysosomes in PTA-treated cells. We also performed immunofluorescence assay and observed that PTA induced co-localization (yellow) of the autophagosomal marker GFP-LC3B (green) with mitochondrial spheroids (MitoTracker red), suggesting that PTA could markedly promote mitophagy (Fig. 6D). Another LysoTracker (red) and MitoTracker (green) colocalization assay was used to characterize the process of autophagosomal proliferation and mitophagy (Fig. 6E). Also, specific 14-3-3ζ knockdown significantly impaired PTA-mediated mitophagy (Fig. 6F, G), resulting in reversing the PTA-dependent stabilization of mitochondrial membrane potential, oxidative stress, and mitochondrial mass (Fig. 6H-J). Taken together, these data indicated that PTA-mediated neuroprotection ensued by targeting 14-3-3ζ-dependent mitophagy.

### PTA suppresses expression of ER stress genes for neuronal survival by targeting 14-3-3ζ

**Figure 7:**
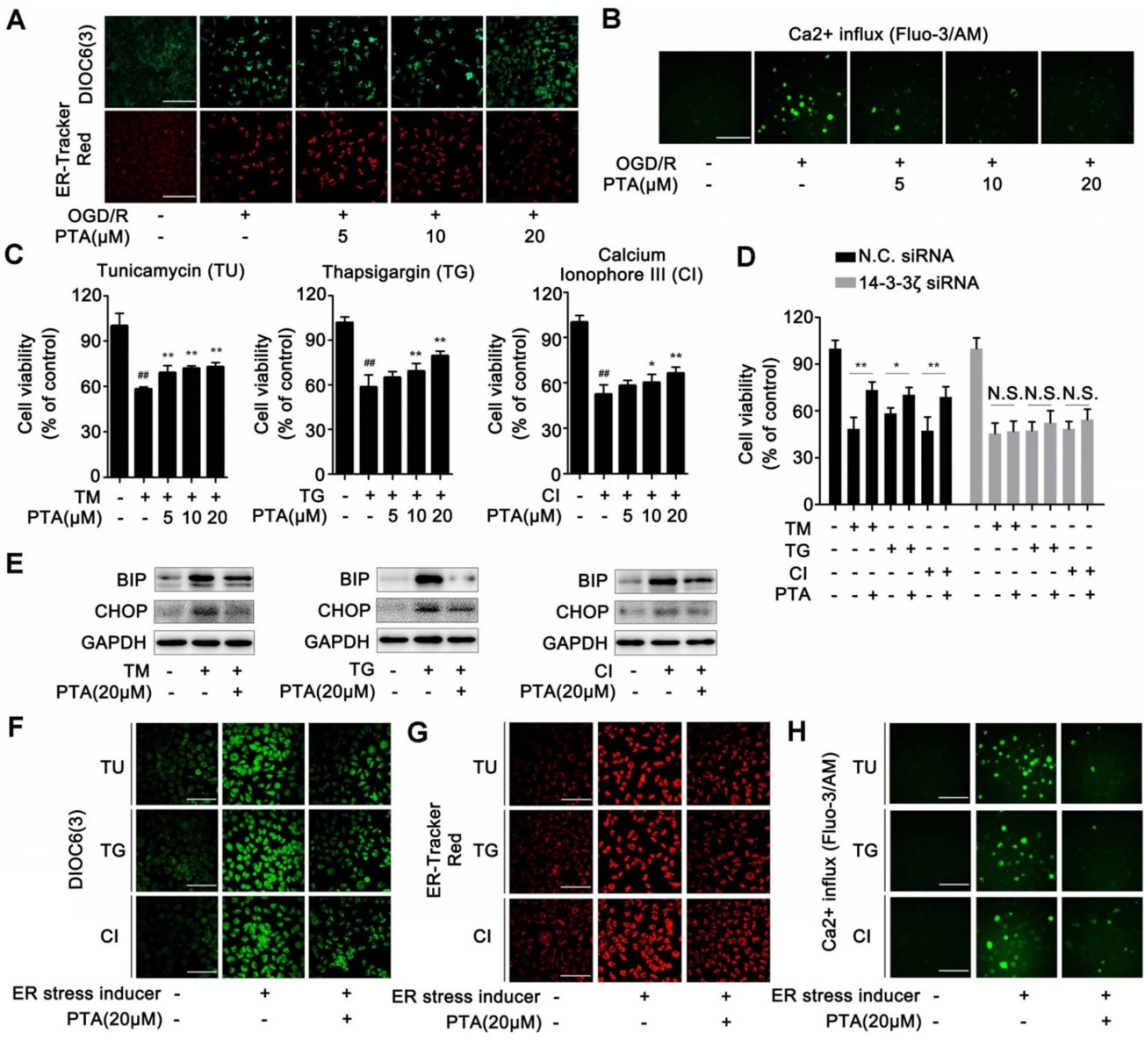
** 14-3-3ζ suppresses ER stress gene expressions for neuronal survival. (A)** PTA inhibited ER stress induced by OGD/R (DIOC6(3) and ER-Tracker Red) in Neuro-2A cells. **(B)** PTA inhibited intracellular calcium overload induced by OGD/R in Neuro-2A cells. **(C)** PTA increased Neuro-2A cell viability against TU, TG, and CI. **(D)** PTA-dependent cell protection against TU, TG or CI was blocked by 14-3-3ζ siRNA in Neuro-2A cells. **(E)** PTA inhibited expressions of ER stress-related proteins BIP and CHOP in Neuro-2A cells. **(F)** PTA inhibited ER stress induced by TU, TG, or CI using ER-Tracker Red staining assay in Neuro-2A cells. **(G)** PTA inhibited ER stress induced by TU, TG, or CI using DIOC6(3) staining assay in Neuro-2A cells. **(H)** PTA inhibited intracellular calcium overload induced by TU, TG, or CI using Ca^2+^-sensitive dye Fluo-3/AM staining assay in Neuro-2A cells. Scale bars = 10 μm.

ER-specific probe ER-Tracker Red and DIOC6(3) staining showed increased numbers of ER-Tracker Red- or DIOC6(3)-positive cells when induced by OGD/R which were significantly inhibited by PTA in a concentration-dependent manner (Fig. 7A). These effects were markedly blocked in 14-3-3ζ siRNA-transfected cells (Fig. S6E). Since calcium homeostasis dysregulation occurs during endoplasmic reticulum stress, we analyzed Ca^2+^ influx using Fluo-3/AM Ca^2+^-sensitive dye and the changes in cytosolic calcium were visualized by confocal laser scanning microscopy. The results showed that OGD/R increased intracellular Ca^2+^ concentration, and this effect was significantly inhibited by PTA (Fig. 7B). Specific 14-3-3ζ knock-down substantially impaired PTA-dependent Ca^2+^ influx inhibition (Fig. S6F). Next, we used ER stress inducers (tunicamycin, TU; thapsigargin, TG or calcium ionophore III, CI) to establish an ER stress-dependent cell injury model to assess the protective effect of PTA. As shown in Fig. 7C, all three ER stress inducers decreased cell viability, which was significantly reversed by PTA. These protective effects were blocked in 14-3-3ζ siRNA-transfected cells (Fig. 7D), suggesting that PTA targeted 14-3-3ζ to suppress ER stress. Further, PTA could significantly inhibit the expression of ER stress-related proteins including BIP and CHOP as detected by Western blotting, indicating a repressive effect of PTA on ER stress (Fig. 7E). ER stress inducers also increased the number of ER-Tracker Red- or DIOC6 (3)-positive cells, which was effectively inhibited by PTA (Fig. 7F, G). Additionally, PTA markedly inhibited ER stress inducer-promoted Ca^2+^ influx (Fig. 7H). In summary, these findings showed that 14-3-3ζ could be a crucial molecular target for PTA-dependent neuroprotection via 14-3-3ζ-dependent ER stress.

## Discussion

Histone H3 PTMs influence genetic expressions and are widely involved in various biological processes. Thus, the development of novel pharmacological strategies for regulating histone H3 PTMs is urgently needed in clinical and translational research. In this study, we identified 14-3-3ζ as a novel transcriptional regulation factor. Its dimerization could mediate histone H3 PTM patterns including acetylation, trimethylation, and phosphorylation, resulting in a significant therapeutic effect on neuronal injury. Furthermore, 14-3-3ζ dimerization could be dynamically controlled by the small-molecule PTA by targeting cysteine189, a previously unexploited allosteric site. These findings revealed the feasibility of editing histone PTM pattern by targeting the transcription regulation factor 14-3-3ζ and demonstrated a distinctive method different from current therapeutic strategies directly acting on histone-modifying enzymes.

Previous investigations showed that 14-3-3 proteins are widely expressed in various diseases and play a pivotal role in cell apoptosis 34, 35. Especially, 14-3-3 biology was reported to be highly associated with adjustment of cerebral circulation as well as protection and functional improvement of neuronal cells 36-38, making it a promising therapeutic target. 14-3-3 proteins typically work as adapter proteins or protein-binding modules that link protein-binding partners and are critical to the regulation of signal transduction for various cell physiological functions. Early studies mainly focused on the development of small-molecule binding to the hotspots on the protein surface for regulating PPIs 39-41. Also, no druggable functional domains have been identified on 14-3-3 structure, making the design and synthesis of specific small-molecule regulators of 14-3-3 a challenge. Here we propose a novel mechanism for allosteric regulation of 14-3-3 with the ultimate goal of exerting a neuroprotective effect by editing histone H3 PTMs.

14-3-3ζ is a small protein and its monomers spontaneously self-assemble as homodimers via conserved sequences 42. 14-3-3ζ dimerization exerts multifunctionality in various pathological processes including cancer cell proliferation, chronic pain, and neuronal injury 43, 44. A key function of 14-3-3ζ dimers is to provide a platform for the interactions of other proteins such as glycoproteins, estrogen receptor, C-Raf, and potassium channel TASK3 45-48. Therefore, regulation of the dynamic process of 14-3-3ζ dimerization through small-molecules holds promise as a therapeutic tool. Due to the lack of a specific catalytic pocket or bioactive domain in 14-3-3ζ structure, it is a challenge to design small-molecules that induce 14-3-3ζ dimerization. Previous explorations focused on the discovery of small-molecules that influence 14-3-3ζ dimerization via stabilization or blockage of PPIs 45; however, there are no reports on strategies for developing small-molecules for regulating 14-3-3ζ dimerization by targeting specific domains. In this study, we discovered a naturally derived small-molecule PTA that promoted 14-3-3ζ dimerization by a novel mechanism. PTA covalently modifies C189 in 14-3-3ζ and induces a conformational change through allosteric effect exposing hydrophobic regions and enabling two monomers to form 14-3-3ζ dimer. To the best of our knowledge, our study is the first report of the chemically-induced, conformational change-mediated dimerization of 14-3-3ζ.

One previous study showed that 14-3-3ζ dimerization contributes to its nuclear localization as well as the interaction with histone H3 31. However, surprisingly, our study suggested that PTA-induced 14-3-3ζ dimerization promoted its cytoplasmic translocation and accumulation. We speculated that the natural and chemically-induced 14-3-3ζ dimers might have distinct conformations. The natural dimer tends to locate in the nucleus and binds to histone H3 by recognizing its phosphorylation site, but the chemically-induced dimer tends to translocate to the cytoplasm. This hypothesis is supported by the fact that chemically induced allosteric domain (27-36aa) is in close proximity to the 14-3-3ζ dimer interface in stereoscopic space, which may indirectly influence the dimer conformation. Furthermore, the dissociation of 14-3-3ζ from histone H3 leads to the exposure of various modification sites as well as PTMs on histone H3. The mutations on these PTM sites promoted the binding of 14-3-3ζ to histone H3. It is possible that histone H3 modification inhibits its association with 14-3-3ζ. Thus, there seems to be a bidirectional feedback loop between histone H3 PTM pattern and its interaction with 14-3-3ζ, indicating that the regulatory mechanisms of 14-3-3 influencing histone H3 are complex and require further extensive investigation.

Our results suggested that 14-3-3ζ existed as a monomer in the nucleus by binding with histone H3. PTA induced dissociation of 14-3-3ζ monomer from histone H3, which translocated to the cytoplasm. Then, 14-3-3ζ monomers interacted with each other to form 14-3-3ζ dimer in the cytoplasm. 14-3-3ζ is known to bind to promoters in autophagy-lysosome pathway such as ATP6V1D, MANBA, and ABCB10 on the genomic DNA. Therefore, 14-3-3ζ translocation resulted in the exposure of specific gene promoters, which could be recognized by transcription factors including YBX1, TIF1β, and E2F3 for transcriptional regulations.

The gene expression profile of PTA treatment was consistent with that of specific 14-3-3ζ siRNA. This finding further supported our hypothesis that small-molecules specifically targeting 14-3-3ζ can partly simulate the transcriptomic changes of genetic 14-3-3ζ knockdown. Notably, there were some discrepancies in gene expression profiles between PTA treatment and 14-3-3ζ siRNA interference. 14-3-3ζ siRNA down-regulated its protein expression and blocked biological functions impacting 14-3-3ζ-regulated gene expression. However, PTA only partially regulated 14-3-3ζ function and PTA treatment for a short time did not affect the interaction of the transcription factors with histone H3. However, further investigation showed that PTA treatment for a longer duration (eg. 4 h) did not induce the interaction between 14-3-3ζ and H3 and some transcription factors even dissociated from histone H3. These findings were almost identical to the effects of 14-3-3ζ knockdown for 48 h, which may partly explain the reason PTA could up- or down-regulate expression of some genes. Taken together, these findings suggest that 14-3-3ζ is a key transcriptional regulation factor responsible for histone H3 PTMs.

ER stress has been implicated as a major cell signaling event in various types of neuronal injury 49, 50. ER stress could activate mitophagy to exert some neuroprotective effects, as confirmed by our observation. PTA exhibited extensive regulatory effects on ER stress and autophagy-lysosome gene expression profiles. Interestingly, we found that PTA-mediated mitophagy activation, as well as ER stress suppression, were blocked in 14-3-3ζ knockdown cells, suggesting that 14-3-3ζ plays a vital role in PTA-dependent neuroprotection. Because ER stress and mitochondrial injury widely occur in the context of neuronal damage, we speculated that small-molecules targeting 14-3-3ζ may show broad-spectrum therapeutic potential in the treatment of various CNS diseases. This hypothesis has been borne out by our observation that PTA protected against neuronal injury induced by 6-OHDa in zebrafish, revealing a potential anti-PD effect.

PTA exerts neuroprotective effects including mitophagy induction and ER stress inhibition by targeting 14-3-3ζ. Therefore, 14-3-3ζ is a crucial arbitrator for PTA-mediated neuroprotection. 14-3-3ζ knockdown could seriously weaken 14-3-3ζ expression, resulting in the loss of PTA-dependent neuroprotective effects. Also, PTA showed weaker neuroprotective effects in 14-3-3ζ siRNA-treated cells. Notably, 14-3-3ζ siRNA-treated cells could still survive in a 14-3-3ζ-independent manner by other alternative pathways. 14-3-3ζ biology is highly complex. Based upon the results of this study, we speculate that PTA only partially regulates biological functions of 14-3-3ζ; however, 14-3-3ζ knockdown can completely block 14-3-3ζ biological functions. Therefore, there may be some 14-3-3ζ functions which are not directly mediated by PTA in our experiments and need to be further investigated in the future.

Overall, our work reveals 14-3-3ζ is a transcription regulation factor which can be used as a druggable target protein and provides a promising therapeutic strategy for neuronal injury by pharmacologically modulating 14-3-3ζ-dependent histone H3 PTM patterns.

## Materials and Methods

### Chemicals and reagents

Hoechst 33258, and calcium ionophore III were obtained from Sigma (St Louis, MO, USA). Acridine orange (AO)/ethidium bromide (EB) double stain assay kit, L-glutathione (Reduced) and tunicamycin were purchased from Solarbio Science & Technology Co. (Beijing, China). Protein A/G-agarose was from Biogot Technology Co. (Nanjing, Jiangsu, China). Lipofectamine RNAiMAX and Lipofectamine 2000 were obtained from Thermo Fisher Scientific (Waltham, MA, USA). Pronase was from Roche Diagnostics GmbH (Mannheim, Germany) and recombinant histone H3 was purchased from Active Motif (CA, USA). LysoTracker, MitoSox, MitoTracker Green, and MitoTracker Red were purchased from Thermo Fisher Scientific (Waltham, Ma, USA). ER-Red and DIOC6 (3) were acquired from Nanjing KeyGen Biotech Co. (Nanjing, Jiangsu, China). Fluo 3-AM special packaging was purchased from Dojindo Laboratorie (Kumamoto-ken, Kyushu, Japan). Thapsigargin was obtained from ACROS Organics (Belgium). LysoSensor^TM^ Yellow/Blue DND-160 (PDMPO) was purchased from Yeasen (Shanghai, China). DO_2_, NaOD, and DCl were acquired from J&K Scientific (Beijing, China). R18 peptide was obtained from Enzo Life Sciences (New York, USA).

### Preparation of protosappanin A (PTA)

Protosappanin A (PTA, C_16_H_16_O_5_) was isolated from *Caesalpinia sappan* L. as described previously 51. The molecular weight of PTA was 272.25and its purity was above 98% by high-performance liquid chromatography.

### BBB trans/ADMET prediction

ChemDraw Professional 16.0 was used to describe all 3D molecular structures. As 3D structure files input to ADMET predictor software, blood-brain barrier permeability and hepatotoxicity of the compounds were estimated based on the internal Quantitative Structure-Activity Relationship (QSAR) model, whereas nephrotoxicity, cardiotoxicity, and neurotoxicity were predicted by self-build QSAR model.

### Evaluation of PTA crossing the BBB

#### Sample preparation

Male Balb/c mice (body weight 20~25 g) were obtained from the Department of Laboratory Animal Science of Peking University Health Science Center and handled according to the Ethical Guidelines of EIACUC-PKU. Mice were kept for 48 h to acclimatize to environment and fasted overnight before treatment.

A total of 6 healthy male Balb/c mice were used for the evaluation of BBB. The brain was collected at sacrifice, immediately after myocardial perfusion, at 0.5, 1, 1.5, and 2 h after intraperitoneal injection of 100 mg/kg PTA in 0.9% saline. Tissue homogenates were prepared in saline using Minilys tissue homogenizer (Bertin Technologies) and stored at -80 °C. Drug-free mice were used for preparing blank tissue homogenates and quality control samples (QC). All samples were vortex mixed for 3 min and centrifuged at 13000 rpm for 30 min. Protein precipitation with acetonitrile was used to prepare samples and the supernatants were evaporated in 200 μL methanol-water. An aliquot of 5 μL of supernatant was analyzed by LC-MS/MS.

#### LC-MS/MS analysis

All analyses were detected on an Agilent triple-quadrupole 6410 mass spectrometer. A linear gradient was employed at a flow rate of 0.4 mL/min, starting from 15‒90% B for 5 min and holding for 6 min at 90% B. Mobile phase comprised of a linear gradient of deionized H_2_O (A) and methanol (B). Qualitative ion pair was *m/z* 271.1→228.9 for PTA.

### Cell culture

Neuro-2A, rat pheochromocytoma (PC12), and human embryonic kidney 293T (HEK293T) cells, obtained from the Peking Union Medical College Cell Bank (Beijing, China), were maintained in high-glucose Dulbecco's Modified Eagle's Medium (DMEM) medium with 10% fetal bovine serum (FBS), 100 μg/mL streptomycin, and 100 U/mL penicillin at 37 °C in 5% CO_2_. Primary cortical neurons were prepared from embryonic day 18 mouse brains. Briefly, cerebral cortices of Kunming mouse embryos were dissected, and the cortex tissues were cut into fragments, followed by incubation in 0.2% trypsin for 20 min at 37 ºC. After the incubation, the cortices were washed in ice-cold HBSS followed by dispersion into single neurons. The isolated cells were seeded on poly-L-lysine pre-coated plates for 4 h, and the medium was replaced by neuro basal medium supplemented with B27 and cultured for 7 days.

### Target validation by cellular thermal shift assay (CETSA)

For the living cell CETSA experiment 24, the cells were treated with PTA at 20 μM for 2 h, and then heated individually at indicated temperatures (42-58 °C) for 3 min followed by addition of KB (30µL) and lysed using 3 cycles of freeze-thawing with liquid nitrogen. The cell lysates were then analyzed by SDS-PAGE and immunoblotted with specific antibody for 14-3-3ζ.

### Mitochondrial mass analysis

The mitochondrial mass analysis was performed using MitoTracker^®^ Red FM (Invitrogen, CA, USA). Briefly, culture supernatants were removed and replaced with medium containing 500 nM MitoTracker^®^ Red FM. Subsequently, the cells were incubated for 40 min at 37 °C in the dark. The fluorescence intensity was detected by a fluorescence spectrophotometer (PerkinElmer EnSpire^TM^, Waltham, MA, USA) under excitation wavelength 581 nm and emission wavelength 644 nm.

### Mitophagy and lysosomal acidity analysis

#### (1) Mitophagy analysis

For MitoTracker-GFP-LC3B dual fluorescent staining, the cells were plated in glass-bottom dishes at 2.0×10^5^ per well one day prior to adenovirus infection. The cells were treated with 1mL of DMEM (10% FBS) containing GFP-LC3B adenovirus. The cells were monitored at 48 h post-infection for green fluorescence (495 nm/519 nm) and further treated with 100 nM MitoTracker Red for red fluorescence (579 nm/599 nm) using confocal laser scanning microscopy (TCS SP8 MP FLIM, Leica, Germany). For MitoTracker-LysoTracker dual fluorescent staining, the cells were plated at 2.0×10^5^ cells/well in glass-bottom dishes and allowed to grow overnight. Subsequently, the cells were incubated at 37 °C with 100 nM MitoTracker Green (495 nm/519 nm) and 100 nM LysoTracker Red (577 nm/590 nm) for 45 minutes and the co-localization of mitochondria and lysosomes were analyzed using confocal laser scanning microscopy (TCS SP8 MP FLIM, Leica, Germany).

#### (2) Lysosomal acidity analysis

For LysoSensor^TM^ Yellow/Blue DND-160 (PDMPO) fluorescent staining, the cell cultures were incubated with 1 μM PDMPO solution for 3 min under 37 °C in the dark and washed three times with PBS.

### Chromatin immunoprecipitation (ChIP) assay

HA-14-3-3ζ expression plasmid was transiently transfected into 1x10^6^ HEK293T for 48 h using Lipofectamine 2000 (Invitrogen, CA, USA) according to the manufacture's introductions. After transfection, the cells were treated with or without PTA (20 μM) for 6h. Subsequently, the proteins were cross-linked with 1% formaldehyde in PBS for 10 min and the reaction was then terminated with 125 mM glycine for 5 min at room temperature. The DNA extraction was performed using the Pierce Magnetic ChIP Kit (Thermo Fisher Scientific, MA, USA) following the manufacturer's instructions. The concentration of DNA was quantified by Qubit® DNA Assay Kit using the Qubit^®^ 2.0 Fluorometer (Life Technologies, CA, USA), and library quality of the DNA was evaluated by Agilent Bioanalyzer 2100 system. DNA fragment libraries were subsequently sequenced on an Illumina Hiseq platform.

For ChIP-seq data analysis, raw sequencing reads passing quality control were aligned to the human reference genome (hg19/GRCh37) using BWA v0.7.12. Model-based analysis of CHIP-Seq (MACS, version 2.1.0) was applied to identify the regions of IP enrichment that pass a *q* value threshold of 0.05 for all data sets. Peak analysis was based on the fold-enrichment of the peaks from different experiments. A peak was determined to be different when the odds ratio between the two groups was more than 2. MEME was used to detect the sequence motif, and TomTom software was performed to annotate the motifs.

### Immunofluorescent analysis

The cells were seeded onto glass coverslips coated with poly-L-lysine for 24 h. Subsequently, the cells were transiently transfected with appropriate plasmids for 48 h. After transfection, the cells were treated with or without PTA (20 μM) as indicated. The cells were fixed in 4% paraformaldehyde for 20 min and then permeabilized with 0.5% Triton X-100 for 30 min. After washing with PBS, the cells were blocked with 5% BSA for 1 h at room temperature, washed 3 times with PBS, and probed with primary antibodies overnight at 4 ºC. The cells were incubated with the secondary antibody conjugated to rabbit Dylight-594 or mouse Alexa Flour-488 for 1 h at room temperature. Images were captured (594/618 nm for Rabbit Dylight-594; 488/519 nm for mouse Alexa Flour-488) using a confocal laser scanning microscope (TCS SP8 MP FLIM, Leica, Germany).

### MS analysis of target proteins

The protein bands with obvious difference upon PTA treatment were isolated and trypsin-digested. The trypsin-digested peptides were analyzed by the LC system equipped with an LTQ Velos pro mass spectrometer (Thermo Fisher Scientific, MA, USA) and an RP-C18AQ column (100 μm id × 15 cm, Michrom Bioresources, USA).

### Determination of PTA-binding site on 14-3-3ζ

PTA was incubated with recombinant 14-3-3ζ protein at 4 °C overnight, and the mixtures were analyzed by SDS-PAGE. The trypsin-digested protein corresponding to 14-3-3ζ was analyzed by EASY-LC II system coupled with an LTQ-Orbitrap velos pro mass spectrometer (Thermo Fisher Scientific, MA, USA) equipped with a nano-electrospray ion source and operated in a data-dependent mode. The LC was equipped with an analytical column (75 μm, 10 cm) packed with 3 μm RP C18. The mobile phase was composed of water (solvent A: 0.1% formic acid in water) and acetonitrile (solvent B: 0.1% formic acid in acetonitrile). Extracted peptides were separated using the following gradient: 2% - 40% B for 70 min; 40% - 95% B for 5 min; 95% B for 20 min (flow rate 300 nL/min). Full scan MS spectra were acquired from *m/z* 350 to 2000 in the Orbitrap analyzer with a resolution of 60,000 (FWHM). MS/MS scans were carried out for the top 15 most abundant precursor ions with charge states ≥ 2 in the linear ion trap analyzer with a CID of 35% collision energy. Proteome Discoverer (1.4) workstation was applied for the elucidation of mass spectrometric data with the SEQUEST search engine (Thermo Fisher Scientific).

### Surface plasmon resonance (SPR) and biolayer interferometry (BLI) analysis

#### (1) SPR analysis

The biomolecular interaction between PTA and 14-3-3ζ was analyzed by SPR using the Biacore T200 system (GE Healthcare, Uppsala, Sweden) at 25°C. Briefly, CM5 sensor chip was activated by using sulpho-NHS/EDC chemistry. The chip was subsequently immobilized with the recombinant human 14-3-3ζ protein at a concentration of 500 μg/mL in sodium acetate, pH 4.0 and then blocked with ethanolamine. Various concentrations of R18 peptide (a phosphor-serine) were injected for method validation at a flow rate of 30 μL/min in PBS-P (10 mM phosphate buffer with 2.7 mM KCl and 137 mM NaCl, 0.05% Surfactant P20 and 5% DMSO) running buffer. All screening compounds were dissolved in DMSO further diluted with PBS-P buffer and analyzed at a final concentration of 50 μM (with 5% DMSO (v/v)). The results were analyzed with the Biacore evaluation software (T200 Version 1.0). The interaction between PTA and 14-3-3ζ was fitted to the steady-state affinity model to obtain results.

#### (2) BLI analysis

The protein interaction between histone H3 and 14-3-3ζ was analyzed by biolayer interferometry (BLI) using Octet RED96e (ForteBio, CA, USA), equipped with super streptavidin biosensors (ForteBio, CA, USA). Briefly, the recombinant histone H3 was biotinylated by incubating with Biotin Reagent (EZ-Link® NHS-Biotin Reagents, Thermo) for 1 h at room temperature. Then the biotinylated histone H3 was immobilized on Super streptavidin biosensors (ForteBio, Menlo Park, CA). The reference sensors were incubated in assay buffer without protein. Both sets of sensors were blocked with 0.1% BSA at room temperature. 25 µM of 14-3-3ζ was added to the sensor and the change in binding was measured over 120 s. Results were analyzed with the ForteBio data analysis software using a double reference subtraction.

### Recombinant 14-3-3ζ protein expression and purification

The DNA sequence encoding 14-3-3ζ was cloned into the *Nde I* restriction sites of the modified pET-28a vector with His-tag. *Escherichia coli* BL21 (DE3) strain was transformed with the recombinant plasmid and grown at 37 °C in Lysogeny broth (LB) medium in the presence of 50 μg/mL kanamycin. After 8 h, protein expression was induced by 0.5 mM isopropyl-β-D-1-thiogalactopyranoside (IPTG) for an additional 8 h at 25 °C. The cells were collected by centrifugation at 6,000 × g for 5 min. For protein purification, the cells were suspended in 40 mL lysis buffer (20 mM Tris-HCl (pH 7.9), 500 mM NaCl), lysed by sonification, and centrifuged at 12000 × g for 15 min at 4 °C. The supernatant was then passed through 0.22 μm syringe filters and loaded onto Ni-NTA resin (CWBIO, Beijing, China), washed with 10 mM imidazole, and eluted with 500 mM imidazole. Proteins were concentrated by centrifugal filtration (Amicon Ultra-0.5, Millipore, USA) with a molecular weight cutoff of 10 kDa. The proteins were further purified through AKTA Avant Chromatography System with gel filtration on Superdex75 Increase 10/300 GL column (GE Healthcare, Chicago, Illinois, USA). The eluted proteins were analyzed by 12% (w/v) SDS/PAGE, and purity was estimated at >95%. The proteins were concentrated and stored in buffer (50 mM sodium phosphate, pH 7.4, 100 mM NaCl, and 30% (v/v) glycerol) at -80 °C.

### Isothermal titration calorimetry (ITC) analysis

The binding affinities and thermodynamics of 14-3-3ζ protein and PTA were determined using a MicroCal PEAQ-ITC (Malvern Instruments, Ltd., UK). Prior to titration, 0.025 mM 14-3-3ζ protein was prepared in Akat buffer (50 mM sodium phosphate, pH 7.4, 100 mM NaCl). PTA was dissolved at 0.5 mM in DMSO. The sample pool (V=280 µL) was prefilled with 0.025 mM 14-3-3ζ protein and then titration with 0.5 mM PTA was performed at 25°C using an initial injection of 0.4 µL followed by 12 successive injections of 2 µL with a 120 s interval.

### STD NMR analysis

STD NMR was performed to investigate small molecule-protein interactions as previously described. Briefly, the NMR spectra were acquired at 25 °C on a Bruker Avance III-600 MHz (proton frequency) spectrometer. The sample contained PTA (500 μM) and the recombinant 14-3-3ζ protein (10 μM) dissolved in phosphate buffer (50 mM sodium phosphate, pH 7.4, 100 mM NaCl) for NMR data acquisition and analyzed by Bruker topspin 2.1.

### 14-3-3ζ dimer formation analysis

#### (1) Crosslinking protein interaction analysis

Chemical crosslinking reagent disuccinimidyl suberate (20 mM HEPES, 100 mM KCl, 1 mM dithiothreitol, pH 8.0) for covalently linking proteins was used. Briefly, 0.025 mM 14-3-3ζ protein was incubated with or without PTA at 25 °C for 8 h. Then, the products were crosslinked by adding disuccinimidyl suberate (1 mM) for 30 min and quenched by adding 50 mM Tris-HCl for 15 min. Finally, the samples were detected using SDS-PAGE followed by silver staining.

#### (2) Analytical ultracentrifugation

Sedimentation equilibration analysis was performed with ProteomeLab XL-I analytical ultracentrifuge (BeckMan, CA, USA). Briefly, 25 μM 14-3-3ζ protein was incubated with or without PTA at 25 °C for 8 h. Then, the products were centrifuged at 20 °C and 60000 rpm for 7 h. The c(s) for each mixture was calculated using SEDPHAT software. Distributions were normalized by area for presentation.

#### (3) Bimolecular fluorescence complementation (BiFC) analysis

The BiFC experiment was performed as previously described 52. GFP (1-155)-N-link-14-3-3ζ and GFP (156-238)-N-link-14-3-3ζ BiFC constructs were subcloned into pcDNA3.1. For optimal fluorophore reconstitution, transiently co-transfected HEK293T cells were incubated for 48 h, followed by treatment with PTA for 3 and 12 hours. Then, the cells were observed (absorption/emission: 394/509 nm) using OLYMPUS IX73 fluorescence microscope (Olympus, Tokyo, Japan).

### Hydrogen-deuterium exchange mass spectrometry (HXMS) analysis

HXMS experiment was performed as described previously 53. Briefly, deuterium labeling was initiated with a 20-fold dilution into D_2_O buffer of a pre-equilibrated (30 min, RT) aliquot of 14-3-3ζ protein and 14-3-3ζ protein with PTA stock solution. The labeling reaction was quenched with the addition of quenching buffer (37.5% [v/v] hydrochloric acid) at indicated times (0.25, 1, 10, 20, 60 and 240 min). Subsequently, samples were injected and online digested by a Waters ENZYMATE BEH pepsin column (2.1×30 mm, 5µm). The peptides were then trapped and desalted on a VanGuard Pre-Column trap (ACQUITY UPLC BEH C18, 1.7 µm) for 3 min, eluted in the trap using 15% acetonitrile at a flow rate of 100 µL/min, and separated using an ACQUITY UPLC BEH C18 column (1.7µm, 1.0×100 mm). Relative deuterium levels of all peptides were calculated by subtracting the mass of the undeuterated control sample from that of the deuterium-labeled sample. All mass spectra were analyzed using DynamX 3.0. Deuterium levels were not corrected for back exchange and thus reported as relative.

### Transient transfection of plasmids and siRNA

The cells were transfected with indicated plasmid vectors using Lipofectamine^®^ 2000 Transfection Reagent (Invitrogen, CA, USA) or transfected with siRNA (Suzhou GenePharma, Jiangsu, China) using Lipofectamine^®^ RNAiMAX in Opti-MEM I Reduced Serum Medium (Invitrogen, CA, USA) for 48-72 h. The transfected cells were used for further experiments. The siRNAs were listed in supplementary Table S1.

### Transcriptome analysis

#### (1) Sample collection and preparation

For 14-3-3ζ knockdown, HEK293 cells were cultured for 24 h and then transfected using Lipofectamine RNAiMAX (Life Technologies) with 14-3-3ζ siRNA according to manufacturer protocol, and the cells were cultured for an additional 48 h. For PTA treatment, cells were incubated for 24 h. Subsequently, cells were harvested and sorted directly into lysis buffer for subsequent RNA extraction. RNA degradation was detected by 1% agarose gels and RNA concentration was measured by Qubit^®^ RNA Assay Kit using Qubit^®^ 2.0 Fluorometer (Life Technologies, Carlsbad, CA, USA). Three µg RNA was served as input for the RNA sample preparations. Sequencing libraries were generated using NEBNext^®^ Ultra™ RNA Library Prep Kit for Illumina^®^ (NEB, Ipswich, MA, USA). The library preparations were sequenced using an Illumina Hiseq platform and 125 bp/150 bp paired-end reads were generated.

#### (2) Data analysis

Clean data were obtained by removing low quality reads from raw data of fastq format, which were processed through an in-house Perl script. Based on the clean data, all downstream analyses were finished with high-quality reads. The reference genome and gene model annotation files were downloaded from the genome website. Index of the reference genome was built by Bowtie v2.2.3 and paired-end clean reads were aligned to the reference genome using TopHat v2.0.12. Furthermore, HTSeq v0.6.1 was used to count the reads number mapped to each gene. FPKM was then calculated based on the length of the gene and reads count (fold change cut-off >1.5) 54-56.

### Bioinformatics analysis

#### (1) DAVID analysis

Differentially expressed genes between the two groups were systematically analyzed using DAVID online software. Briefly, on accessing the DAVID website (https://david.ncifcrf.gov/), Gene Functional Classification module was chosen, followed by copying the names of differentially expressed genes into 'box A' and selecting the official gene symbol as the gene identifier type. According to DAVID analysis, differentially expressed genes were rich in this endoplasmic reticulum and lysosome signaling pathways. The statistical analysis of the genes covered in these two signaling pathways was performed using MetaboAnalyst online software (http://www.metaboanalyst.ca/).

#### (2) STRING analysis

Network pathway for functional and protein interaction analysis of 14-3-3ζ vs histone H3 was carried out using STRING 10.5 database (http://string-db.org/), which currently contains approximately 9.6 million proteins from 2031 complete genomes.

### Zebrafish swimming performance analysis

#### (1) Locomotion behavioral test of zebrafish with 6-hydroxydopamine model

Zebrafish has been widely used as an *in vivo* model for testing neurological disorders. In brief, zebrafish larvae at 3 days post-fertilization (dpf) were randomly transferred to 24-well plates with 10 larvae per well. The larvae were co-incubated with 250 μM 6-hydroxydopamine (6-OHDA) and different drugs at specified concentrations till 7 dpf. At the end, zebrafish larvae were transferred to 48-well plate (1 fish/well). The swimming pattern and total distance traveled of each larva were recorded for 10 min using a video tracking system (Viewpoint ZebraLab, Lyon, France).

#### (2) Locomotion behavioral test of zebrafish with acute cerebral hypoxia injury

Zebrafish larvae at 6 days post-fertilization (dpf) were transferred to a sealed container with fish water. The box was filled with high purity nitrogen gas until the dissolved oxygen concentration reduced to 0.5 mg/L. After 1 min, the larvae with acute cerebral hypoxia injury were transferred to 24-well plates with 10 larvae per well. The larvae were incubated with different drugs at specified concentrations. After 24 h, zebrafish larvae were transferred to 48-well plate (1 fish/well). The swimming pattern and total distance traveled of each larva were recorded for 10 min using a video tracking system (Viewpoint ZebraLab, Lyon, France).

### Animal studies

Male SD rats (250-280 g) were obtained from VITAL RIVER Laboratories (Beijing, China). Rats were kept at 25 ± 1 °C under constant dark and light cycles. Animals were provided with water and standard pellet diet freely throughout the experiment. They received good care under the guidelines of CPCSEA (Committee for Control and Supervision of Experiments on Animals). For the establishment of cerebral ischemia/reperfusion injury rat model, right middle cerebral artery occlusion (MCAO) was performed with an intraluminal silicon-coated filament 57. Drugs were administered orally 1 h before MCAO. Ischemia was induced for 1 h and the suture was withdrawn for reperfusion. 24 h after the surgery, neurobehavioral deficits were graded on a 5-point scale, which was modified from the Longa score. Then the rats were sacrificed, and the brains were dissected out immediately. Additionally, the cerebral infarct size was assessed by 2, 3, 5- triphenyltetrazolium chloride (TTC) staining 58.

OGD/R, MTT, LDH release, Hoechst 33258 staining, AO/EB staining, Western blotting, transmission electron microscope, tryptophan fluorescence quenching study, Co-IP, DARTS, mitochondrial depolarization, and CD analysis assays were performed as previously described 59-60.

### Statistical analysis

All experiments were performed at least three times in triplicate. Statistical data were expressed as means ± standard deviation (S.D.). Comparisons between different groups were carried out with Student's t-test and analysis of variance (ANOVA) as appropriate. Values of *P* < 0.05 were considered to be statistically significant.

## Supplementary Material

Supplementary figures and tables.

## Abbreviations

PTM: histone post-translational modifications
HDACs: histone deacetylases
HATs: histone acetyltransferases
HDMs: histone demethylases
PPIs: protein-protein interactions
PTA: protosappanin A
ER: endoplasmic reticulum
SPR: surface plasmon resonance
CETSA: cellular thermal shift assay
LC-MS/MS: LTQ-Orbitrap Velos pro mass spectrometry
DARTS: drug affinity responsive target stability
NMR: nuclear magnetic resonance
STD: saturation transfer difference
ITC: isothermal titration calorimetry
GFP: green fluorescent protein
HDX-MS: hydrogen/deuterium exchange mass spectrometry
Co-IP: co-immunoprecipitation
