# Supplementary material for: Allosteric regulation of protein 14-3-3ζ scaffold by small-molecule editing modulates histone H3 post-translational modifications
Source: Theranostics. 2020 Jan 1;10(2):797–815. doi: 10.7150/thno.38483 (PMC6929985; doi:10.7150/thno.38483)
Supplement: Supplementary file 1 — Supplementary figures and tables. [file thnov10p0797s1.pdf]

Supplementary tables for methods

Table S1. siRNA sequences

**14-3-3ζ(h)**	5'-3' (sense)	CGUCUCAAGUAUUGAACAATT
**siRNA**	5'-3' (antisense)	UUGUUCAAUACUUGAGACGTT
**14-3-3ζ(h)**	5'-3' (sense)	CACGCUAAUAAUGCAAUUATT
**siRNA**	5'-3' (antisense)	UAAUUGCAUUAUUAGCGUGTT
**14-3-3ζ(r)**	5'-3' (sense)	GGAGCCCGUAGGUCAUCUUTT
**siRNA**	5'-3' (antisense)	AAGAUGACCUACGGGCUCCTT
**14-3-3ζ(r)**	5'-3' (sense)	GCCCUCAACUUCUCUGUGUTT
**siRNA**	5'-3' (antisense)	ACACAGAGAAGUUGAGGGCTT
**14-3-3ζ(m)**	5'-3' (sense)	CGCUAAUAAUGCAGUUACUTT
**siRNA**	5'-3' (antisense)	AGUAACUGCAUUAUUAGCGTT
**14-3-3ζ(m)**	5'-3' (sense)	GGCUUAUUCACAUGCAUUATT
**siRNA**	5'-3' (antisense)	UAAUGCAUGUGAAUAAGCCTT

Supplementary Figures

Figure S1

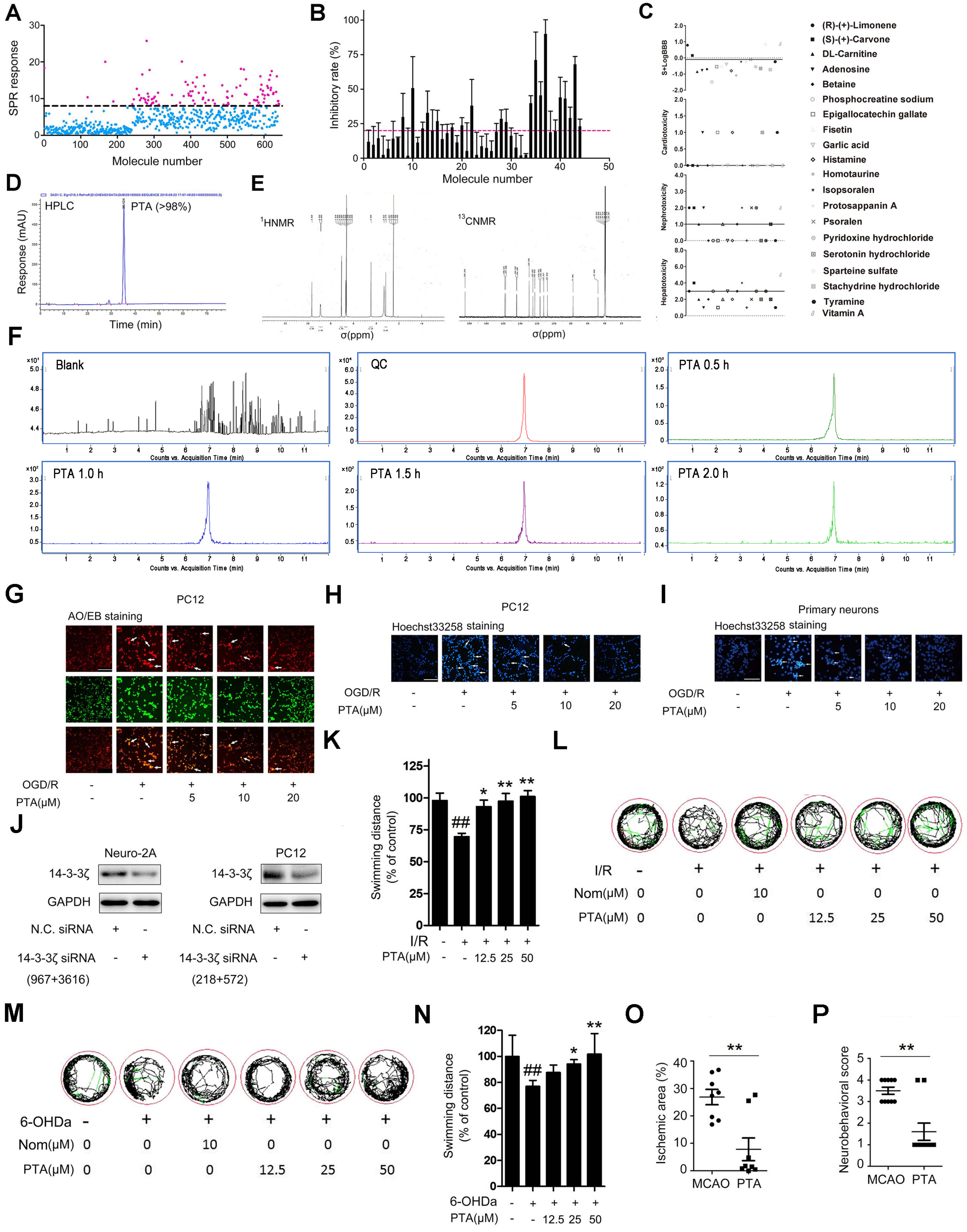

Figure S1. PTA is identified as a small-molecule targeting 14-3-3ζ with neuroprotective effect

(A) Surface plasmon resonance (SPR) screening for 14-3-3ζ-binding small molecules. (B) Neuroprotective small molecules were screened using oxygen and glucose deprivation/reperfusion (OGD/R)-induced Neuro-2A cells. (C) The blood-brain barrier permeability and potential toxicity of 19 molecules. (D) HPLC profile of PTA. (E) ^{1}H and ^{13}C NMR spectra of PTA. (F) Evaluation of PTA passing blood-brain barrier by LC-MS/MS analysis in negative mode (G) AO/EB staining for PC12 cells. (H) Hoechst 33258 staining for PC12 cells. (I) Hoechst 33258 staining for primary cultured neurons. (G) siRNA knock-down for 14-3-3ζ in Neuro-2A and PC12 cells. (K and L) Swimming performance analysis for PTA neuroprotection in ischemia/reperfusion-induced zebrafishes. (M and N) Swimming performance analysis for PTA neuroprotection in 6-OHDa-induced zebrafishes. (O) Cerebral ischemic area evaluation for PTA neuroprotection in MCAO rat model. (P) Neurobehavioral score evaluation for PTA neuroprotection in MCAO rat model.

Figure S2

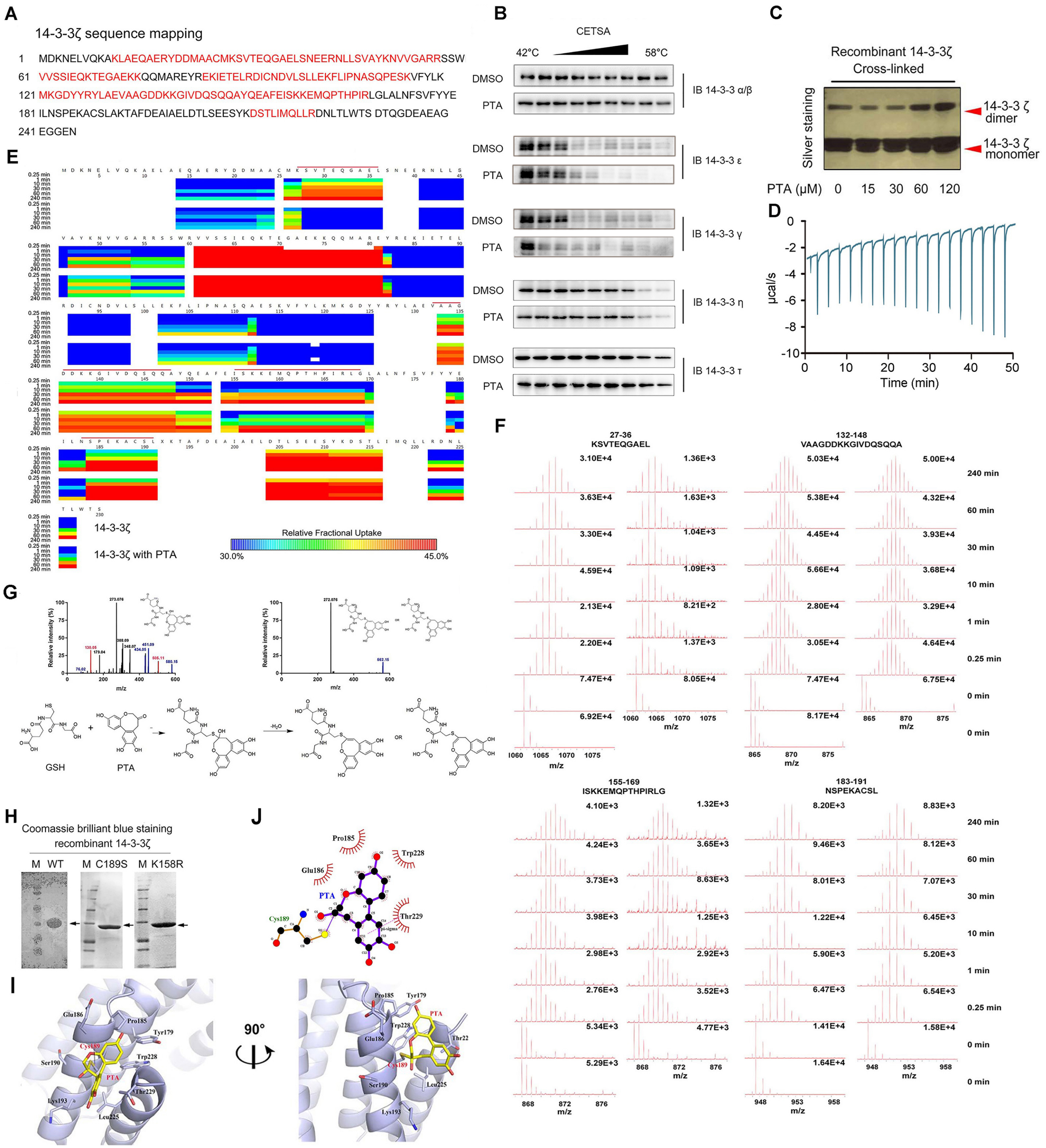
A
14-3-3ζ sequence mapping
1 MDKNELVQKAKLAEQAERYDDMAACMKSVTEQGAELSNEERNLLSVAYKNVVGARRSSW
61 VVSSIEQKTEGAEKKQQMAREYREKIETELRDICNDVLSLLEKFLIPNASQPESKVFYLK
121 MKGDYYRYLAEVAAGDDKKGIVDQSQQAYQEAFEISKKEMQPTHPIRLGLALNFSVFYYE
181 ILNSPEKACSLAKTAFDEAIAELDTLSEESYKDSTLIMQLLRDNLTLWTS DTQGDEAEAG
241 EGGEN
B
CETSA
42°C
58°C
DMSO
PTA
IB 14-3-3 α/β
IB 14-3-3 ε
IB 14-3-3 γ
IB 14-3-3 η
IB 14-3-3 τ
C
Recombinant 14-3-3ζ
Cross-linked
Silver staining
14-3-3 ζ dimer
14-3-3 ζ monomer
PTA (μM) 0 15 30 60 120
D
μcal/s
Time (min)
E
14-3-3ζ
14-3-3ζ with PTA
Relative Fractional Uptake
30.0%
45.0%
F
27-36
KSVTEQGAEL
132-148
VAAGDDKKGIVDQSQQA
155-169
ISKKEMQPTHPIRLG
183-191
NSPEKACSL
240 min
60 min
30 min
10 min
1 min
0.25 min
0 min
m/z
G
Relative intensity (%)
m/z
GSH
PTA
-H₂O
OR
H
Coomassie brilliant blue staining
recombinant 14-3-3ζ
M WT
M C189S
M K158R
I
90°
J
PTA
Cys189
Glu186
Pro185
Trp228
Thr229
Tyr179
Ser190
Lys193
Leu225

Figure S2. PTA promotes 14-3-3ζ dimerization by allosterically regulating the dimer interface

(A) The red marked 14-3-3ζ peptides were identified by LC-MS/MS. (B) CETSA analysis of endogenous 14-3-3α/β, 14-3-3ε, 14-3-3γ, 14-3-3η and 14-3-3τ in PC12 cells. (C) Silver stain analysis for PTA-dependent 14-3-3ζ dimerization. (D) ITC analysis for PTA-dependent 14-3-3ζ dimerization. (E) Representative heat map of deuterium uptake levels of identified peptides from 14-3-3ζ. Deuterium incorporation over 0.25, 1, 10, 30, 60, and 240 min is indicated by color-coded blocks underlining amino acid sequence. Gradient color band shows HDX levels. (F) Mass spectrum of 14-3-3ζ protein with important changes in deuteration level upon interaction with PTA. (G) LC-MS/MS analysis of PTA-binding to GSH and reaction mechanism of PTA with thiol in GSH. (H) Recombinant 14-3-3ζ (WT, C189S, K158R) proteins were prokaryotic expressed in *E.coli* and purified. (I) Interactions of PTA with amino acid residues in 14-3-3ζ were shown in PyMol 3D structure. (J) 2D of PTA-14-3-3ζ protein interaction diagram generated by LigPlus.

Figure S3

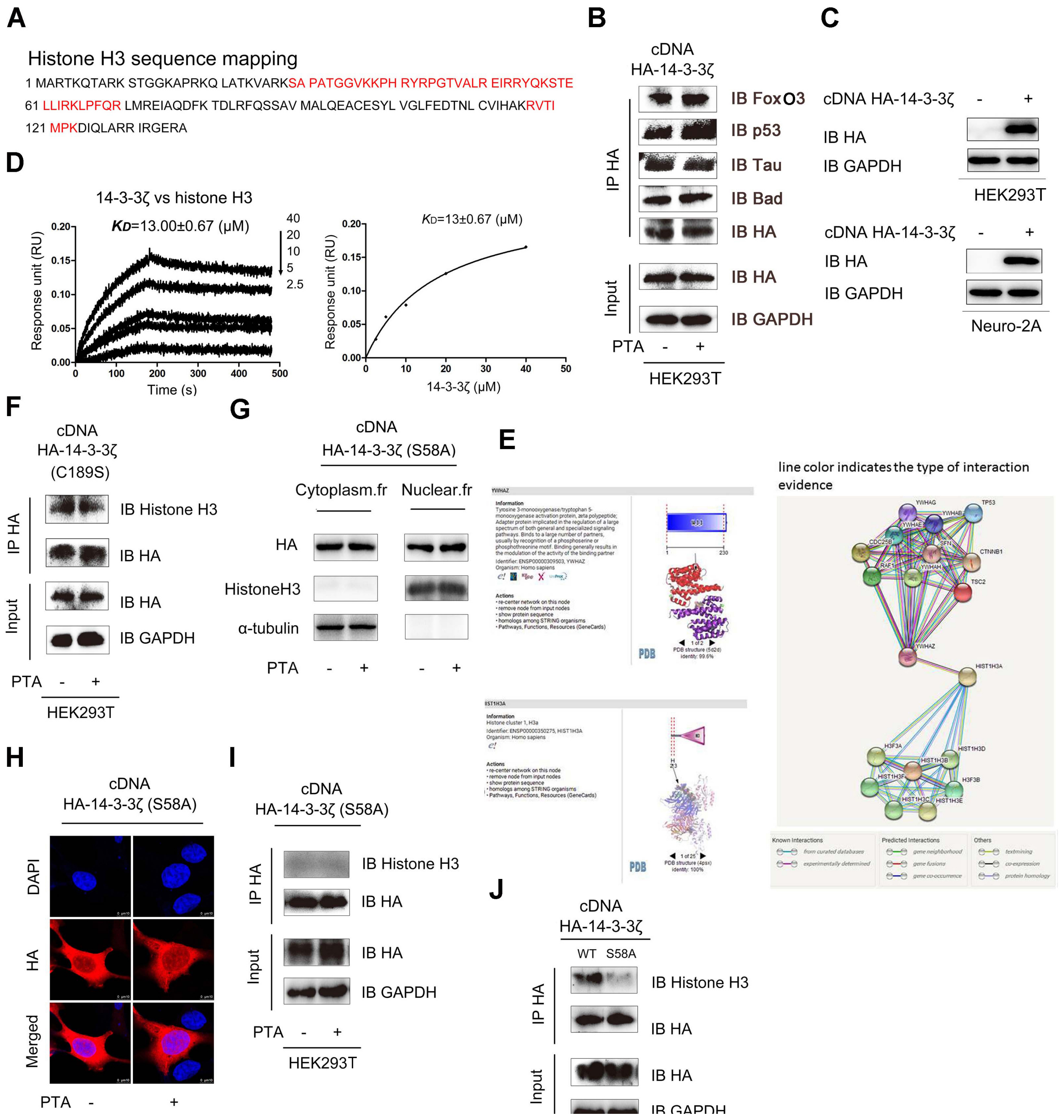

Figure S3. Selective regulation of 14-3-3ζ binding with histone H3 via targeting C189.

(A) Red marked histone H3 peptides were identified by LC-MS/MS. (B) PTA did not mediate interactions of 14-3-3ζ with FoxO3, p53, Tau and Bad. (C) HA-tagged 14-3-3ζ was highly expressed in Neuro-2A and HEK293T cells. (D) Interaction of 14-3-3ζ with histone H3 was detected by biolayer interferometry (BLI) analysis. (E) Interaction of 14-3-3ζ with histone H3 was investigated by STRING. (F) 14-3-3ζ (C189S) interaction with histone H3 was not affected by PTA in HA-tagged 14-3-3ζ transfected HEK293T cells. (G) S58A mutation inhibited PTA-induced cytoplasmic translocation of 14-3-3ζ. (H) S58A mutation prevented PTA-dependent cytoplasmic localization of 14-3-3ζ (scale bars = 10 μm). (I) S58A mutation blocked PTA-dependent interaction of 14-3-3ζ with histone H3. (J) S58A mutation regulated interaction of 14-3-3ζ with histone H3.

Figure S4

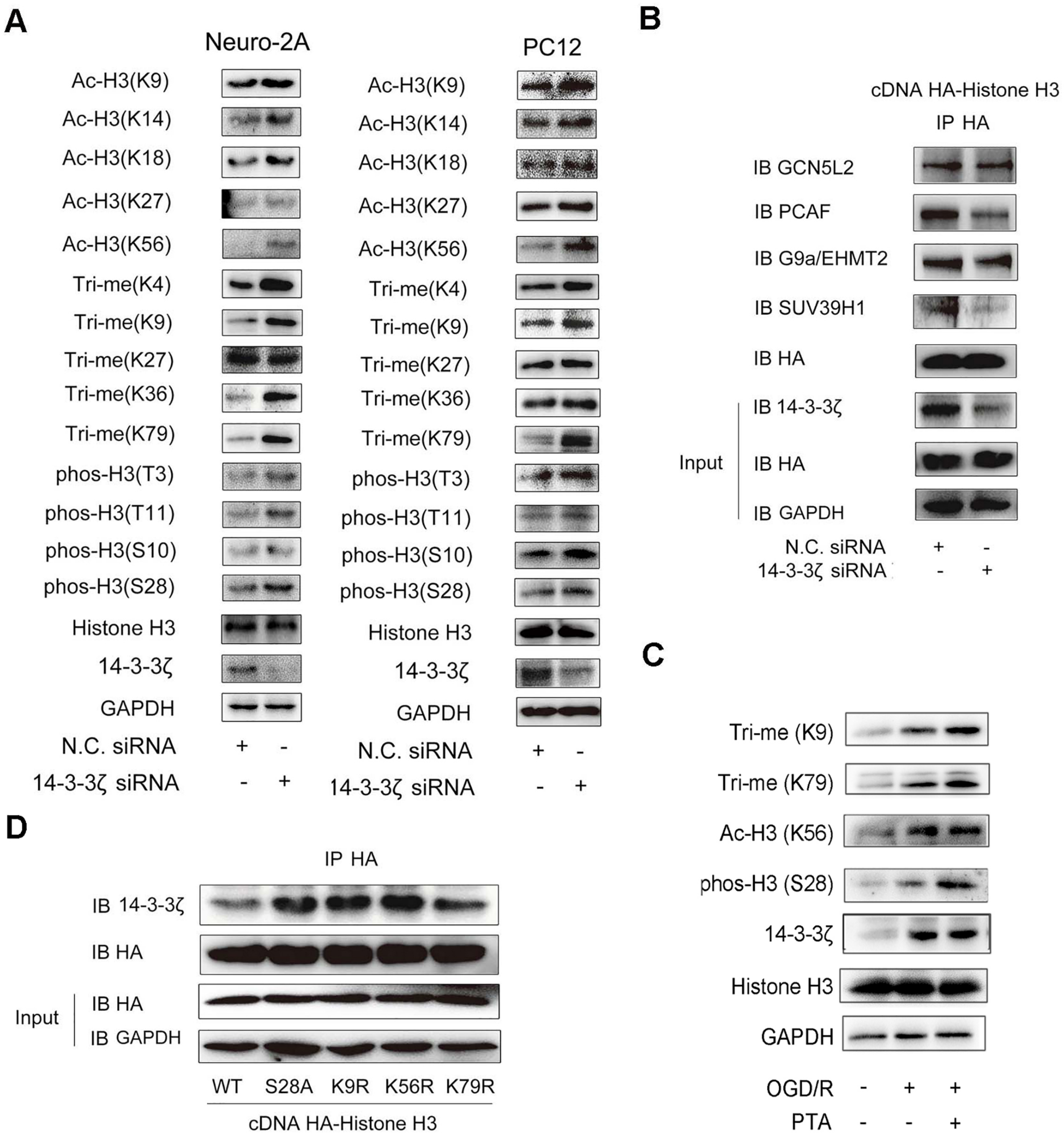

Figure S4. 14-3-3ζ knockdown induces histone H3 PTMs pattern change.

(A) siRNA 14-3-3ζ regulated multiple post-translational modifications on histone H3. (B) siRNA 14-3-3ζ did not show obvious effect on interaction of histone acetyltransferases (HATs) and histone methyltransferases with histone H3. (C) PTA could regulate acetylation, trimethylation and phosphorylation levels of 14-3-3ζ in OGD/R model. (D) Modification site mutations promoted interaction of 14-3-3ζ with histone H3.

Figure S5

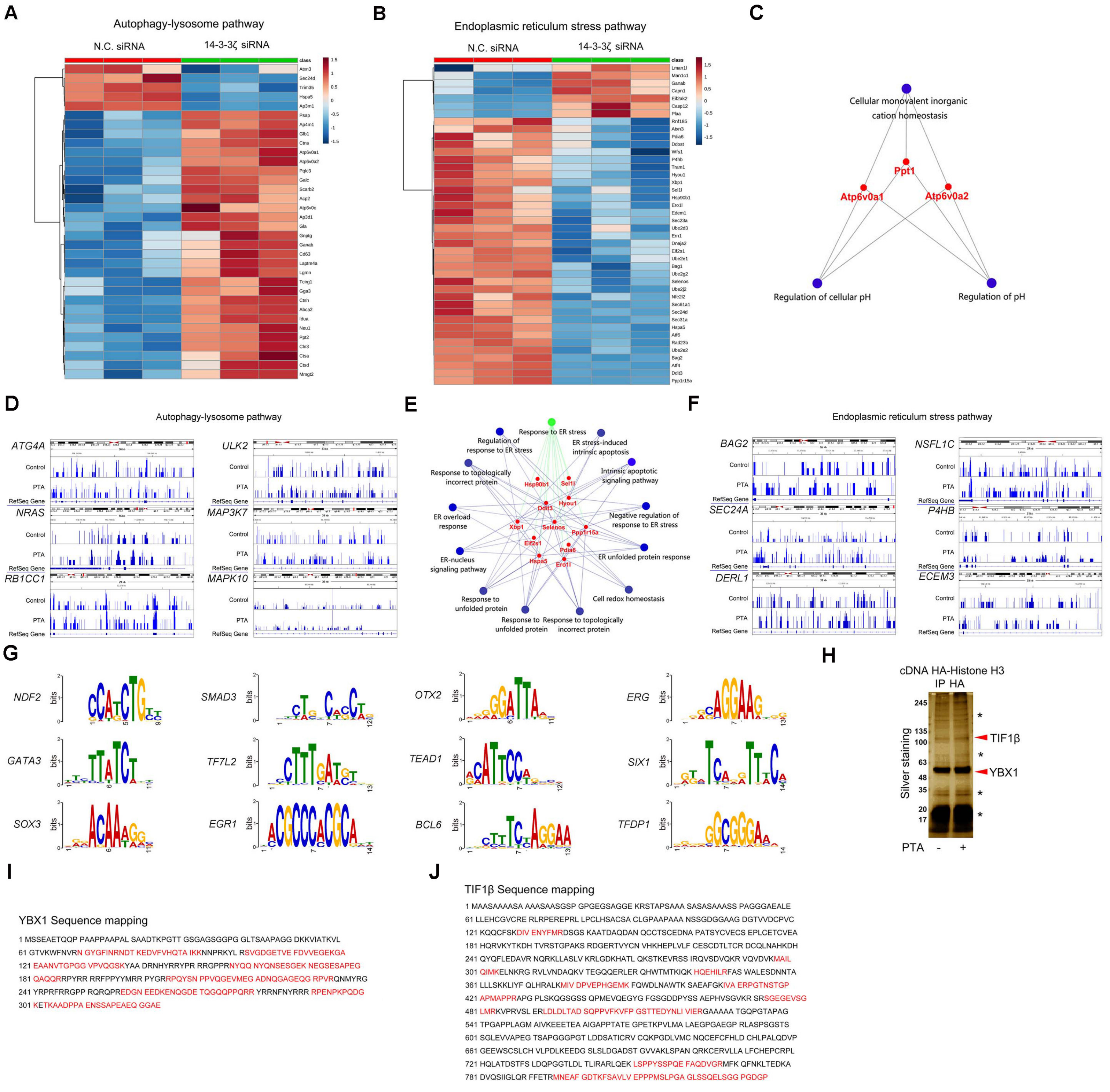

Figure S5. PTA affects gene expressions on autophagy-lysosome and endoplasmic reticulum stress pathways.

(A) Heat map of gene expression changes to core autophagy-lysosome pathway after treatment with siRNA 14-3-3ζ. (B) Heat map of gene expression changes to core endoplasmic reticulum stress pathway after treatment

with siRNA 14-3-3ζ. (C) Biological process analysis of autophagy-lysosome pathway-related transcriptional changes after treatment with PTA at 20 μM. (D) Example IGV screenshots for autophagy-lysosome pathway-related genes were shown. (E) Biological process analysis of endoplasmic reticulum stress pathway-related transcriptional changes after treatment with PTA at 20 μM. (F) Example IGV screenshots for endoplasmic reticulum stress pathway-related genes were shown. (G) TomTom analysis of conserved motifs. (H) Co-immunoprecipitation (Co-IP) was performed with anti-HA antibody followed by silver staining and LC-MS/MS analysis for specific protein bands. HEK293T cells transfected with HA-tagged histone H3 vector were treated with 20 μM PTA or vehicle. (I) Red marked *YBX1* peptides were identified by LC-MS/MS. (J) Red-marked *TIF1β* peptides were identified by LC-MS/MS.

Figure S6

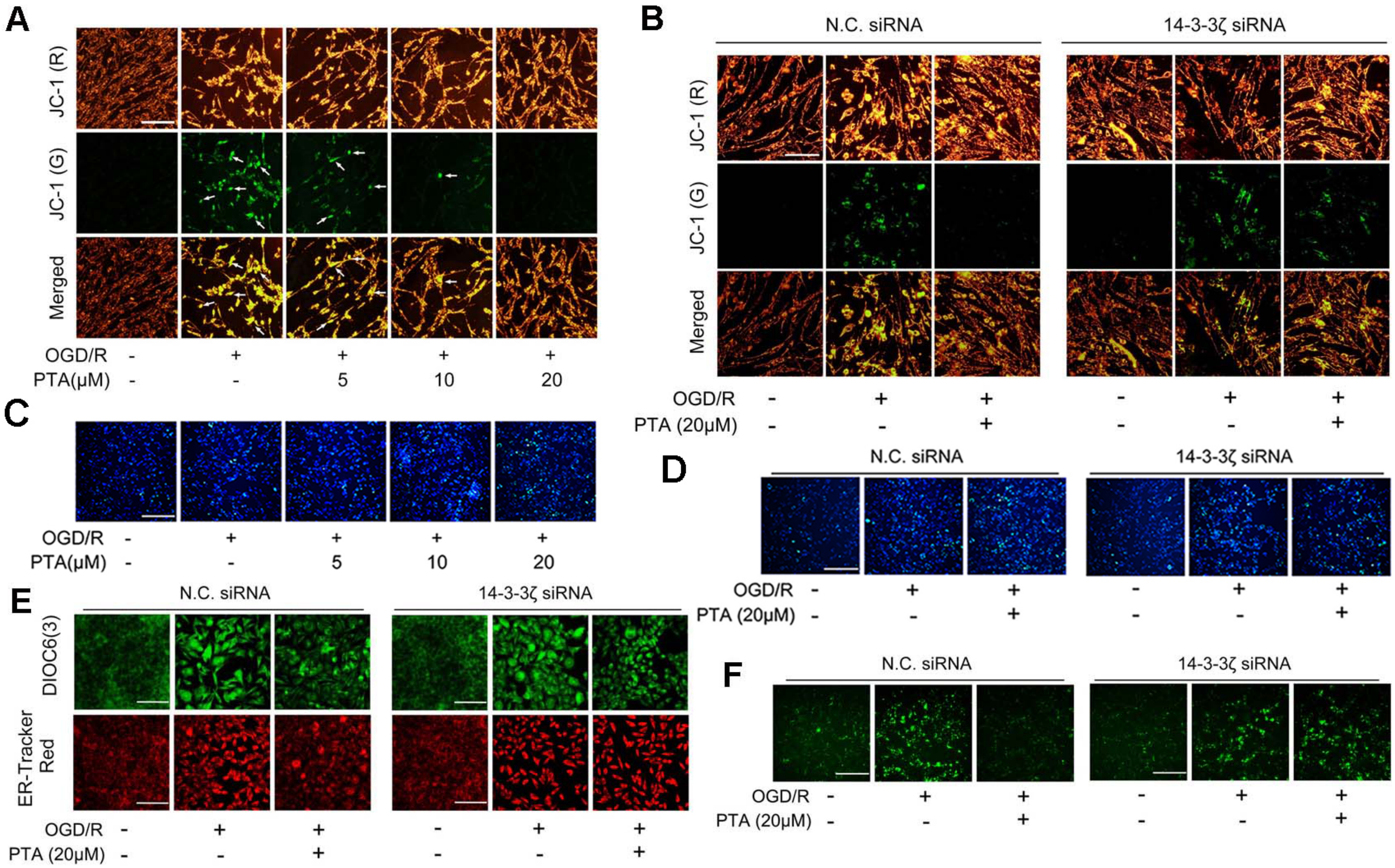

Figure S6. PTA exerts neuroprotection via 14-3-3ζ-dependent mitophagy and ER stress pathways.

(A) Mitochondrial depolarization was inhibited by PTA using JC-1 staining. (B) PTA-dependent inhibition on mitochondrial depolarization was blocked in 14-3-3ζ siRNA-transfected Neuro-2A cells. (C) PTA increased pH of lysosome in OGD/R-induced Neuro-2A cells (LysoSensorTM Yellow/Blue). (D) PTA-dependent increase on pH of lysosome was blocked in 14-3-3ζ siRNA-transfected Neuro-2A cells. (E) PTA-dependent inhibition on ER stress-induced by OGD/R was blocked in 14-3-3ζ siRNA-transfected Neuro-2A cells. (F) PTA-dependent inhibition on intracellular calcium overload-induced by OGD/R was blocked in 14-3-3ζ siRNA-transfected Neuro-2A cells.

Source data

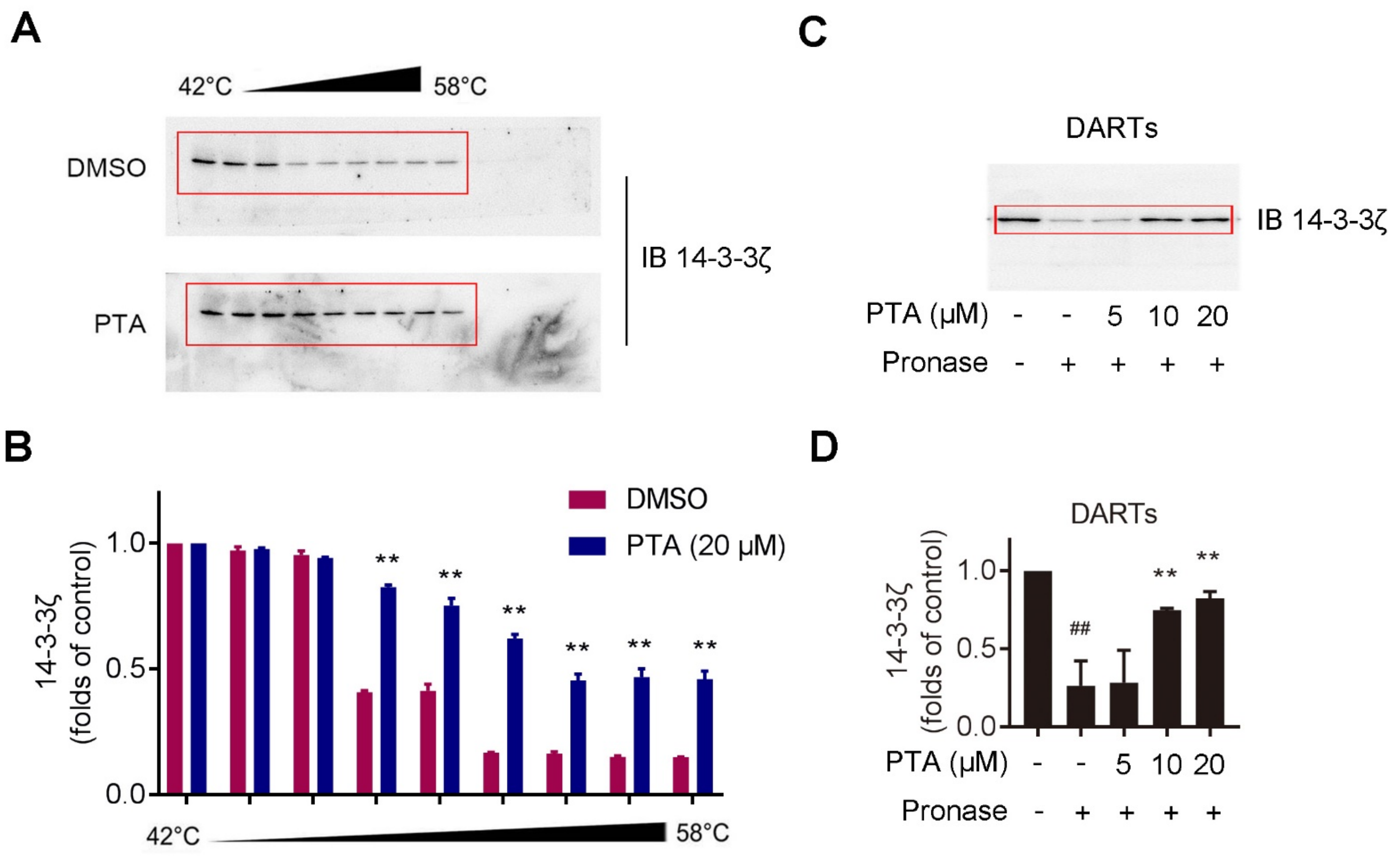

(A and B) Full western blots and quantification of CETSA analysis for 14-3-3ζ. Related to Figure 1I. (C and D) Full western blots and quantification of DARTs analysis for 14-3-3ζ. Related to Figure 1J.

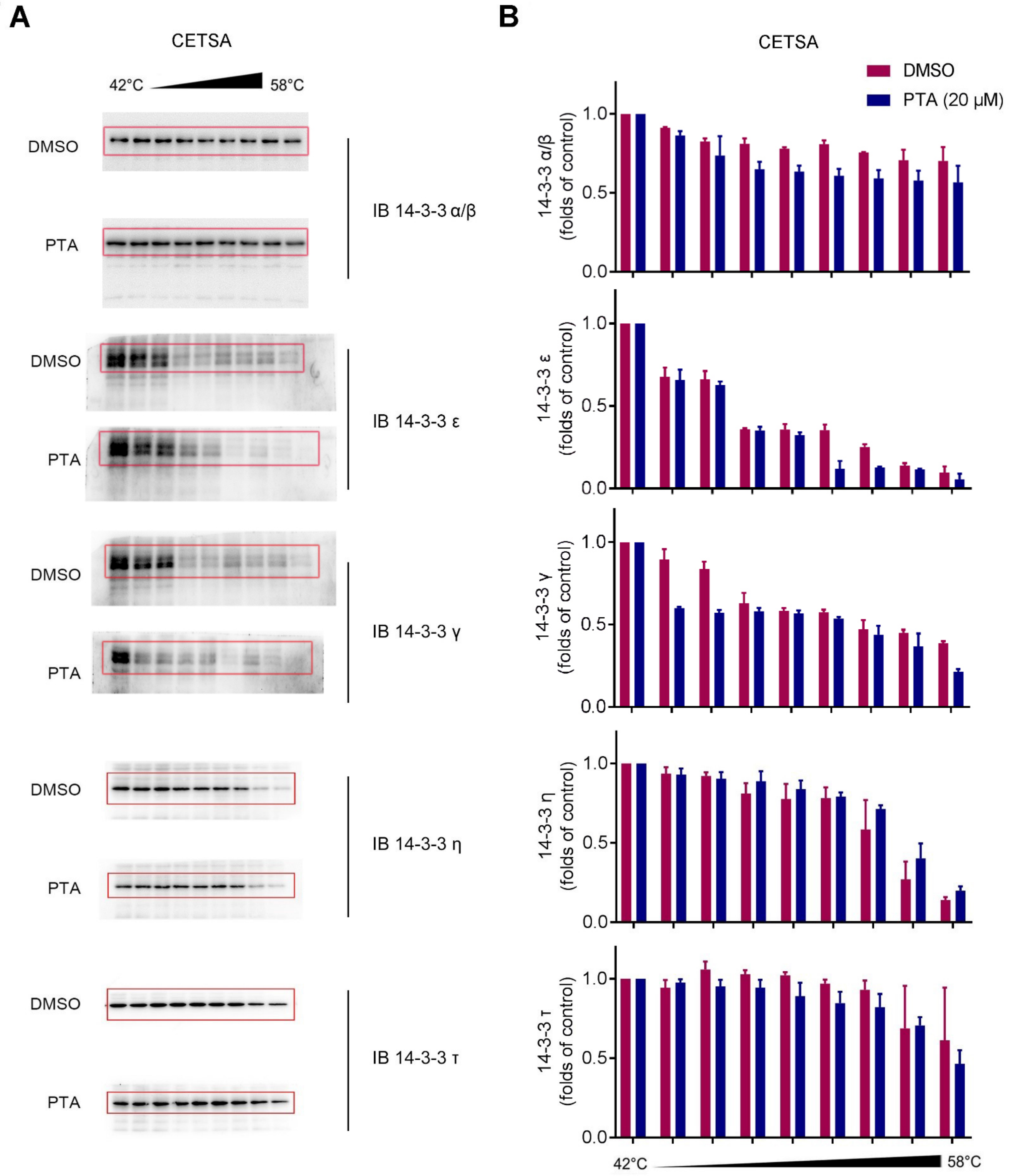

96

97 (A and B) Full western blots and quantification of CETSA analysis for 14-3-3α/β, 14-3-3ε, 14-3-3γ, 14-3-3η
98 and 14-3-3τ. Related to Figure S2C.

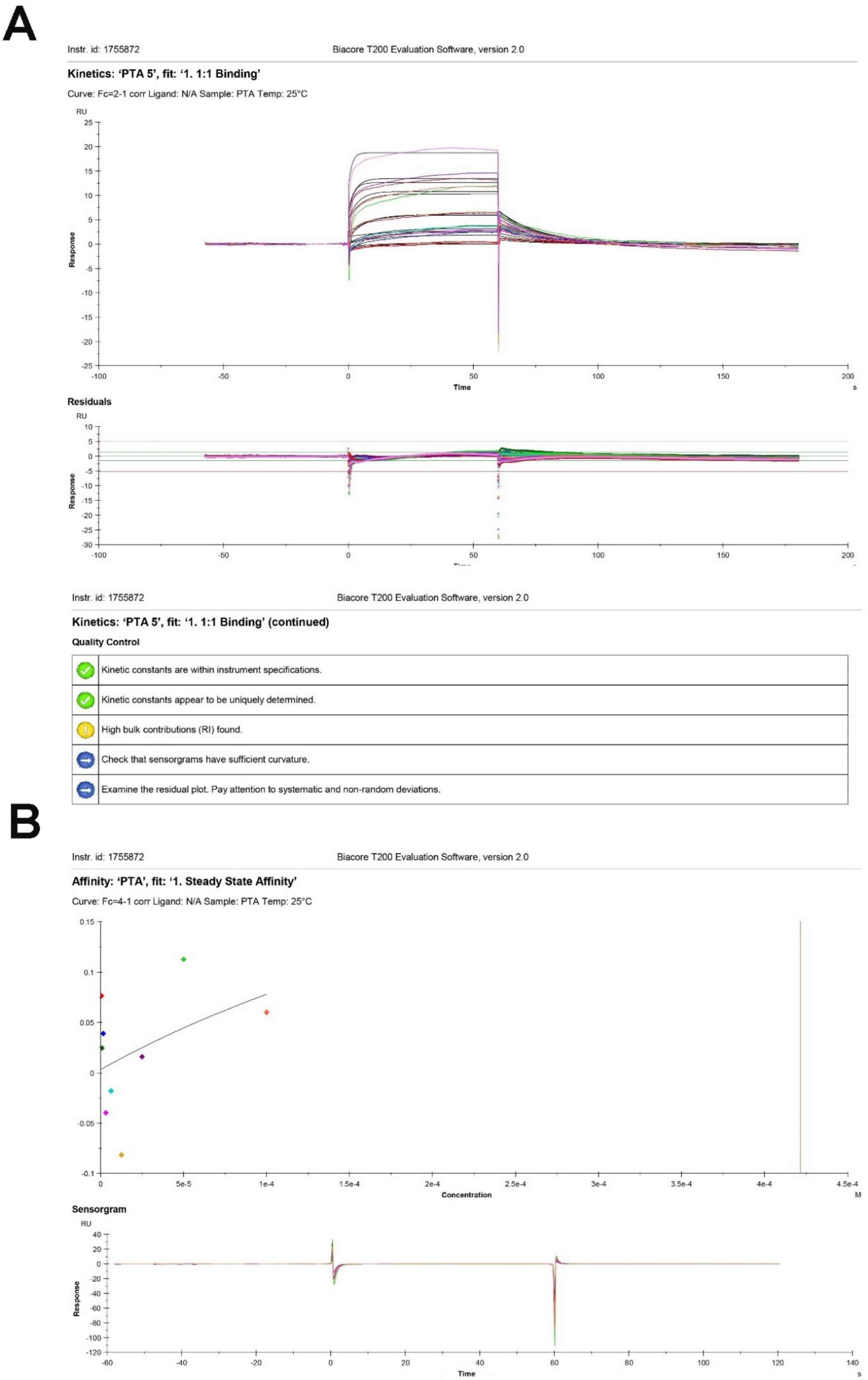

(A) Fitted curves and quality control of SPR analysis. (B) Result of SPR analysis for non-interacted protein.

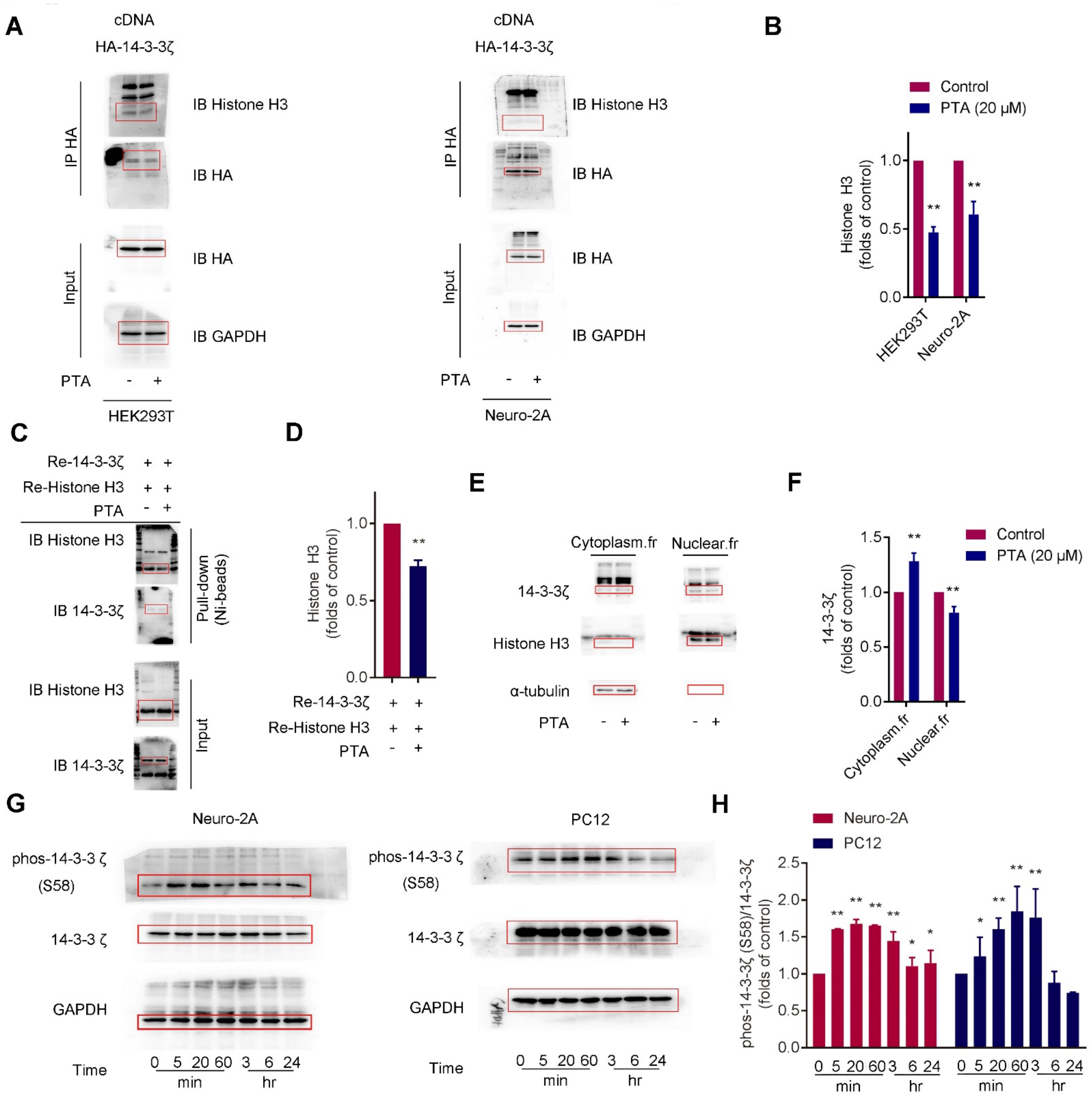

(A and B) Full western blots and quantification analysis. 14-3-3ζ interaction with histone H3 was inhibited by PTA in HA-tagged 14-3-3ζ-transfected HEK293T and Neuro-2A cells. Related to Figure 3B. (C and D) Full western blots and quantification analysis. Recombinant 14-3-3ζ protein interaction with recombinant histone H3 protein was inhibited by PTA. Related to Figure 3C. (E and F) Full western blots and quantification analysis. Cytoplasmic translocation of 14-3-3ζ from the nucleus was promoted by PTA. Related to Figure 3D. (G and H) Full western blots and quantification analysis. Phosphorylation levels of 14-3-3ζ (S58) were increased by PTA. Related to Figure 3G.

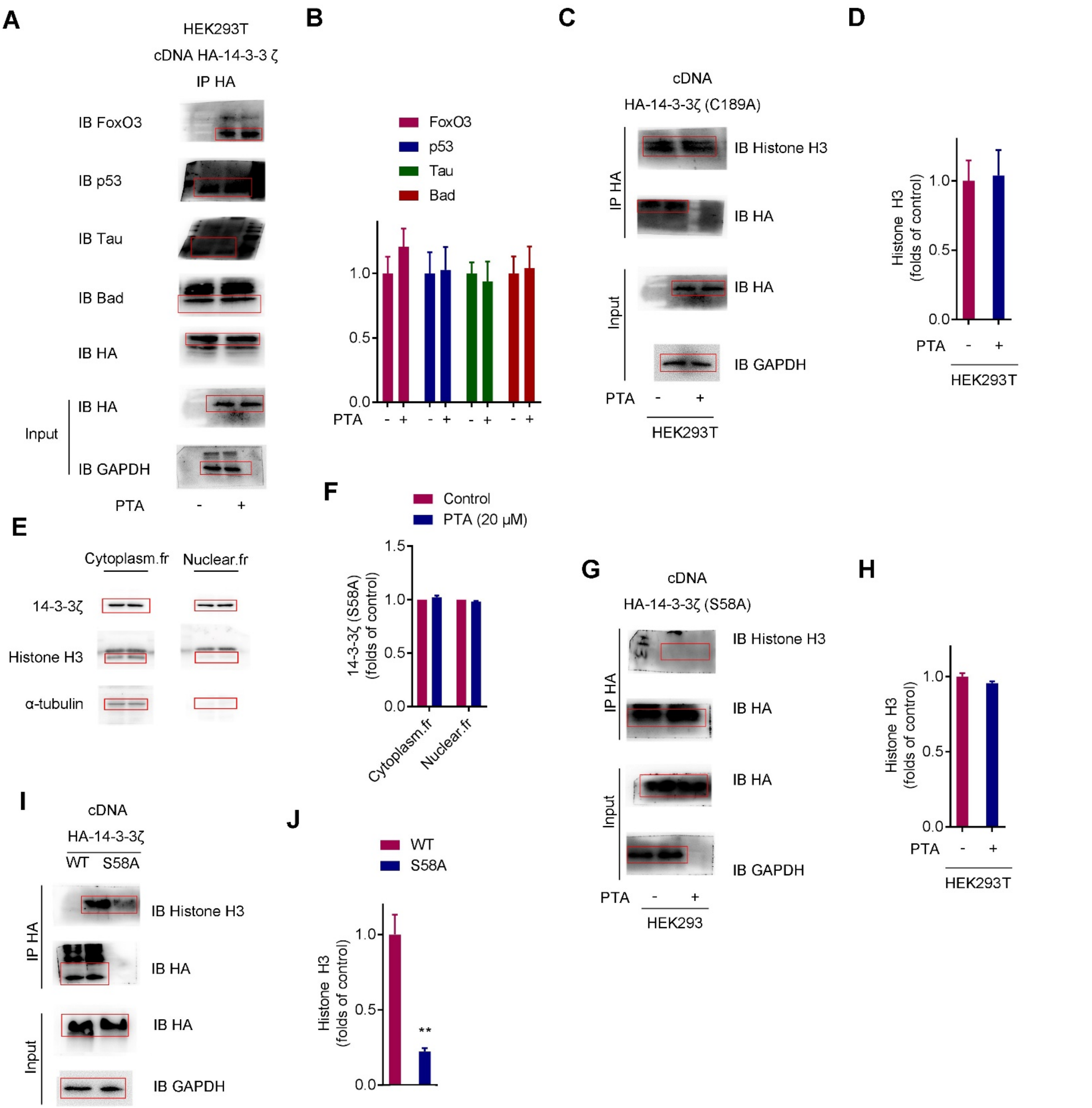

(A and B) Full western blots and quantification analysis. PTA did not mediate interactions of 14-3-3ζ with FoxO3, p53, Tau and Bad. Related to Figure S3B. (C and D) Full western blots and quantification analysis. 14-3-3ζ (C189S) interaction with histone H3 was not affected by PTA in HA-tagged 14-3-3ζ transfected HEK293T cells. Related to Figure S3F. (E and F) Full western blots and quantification analysis. S58A mutation inhibited PTA-induced cytoplasmic translocation of 14-3-3ζ. Related to Figure S3G. (G and H) Full western blots and quantification analysis. S58A mutation blocked PTA-dependent interaction of 14-3-3ζ with histone H3. Related

to Figure S3I. (I and J) Full western blots and quantification analysis. S58A mutation regulated interaction of 14-3-3ζ with histone H3. Related to Figure S3J.

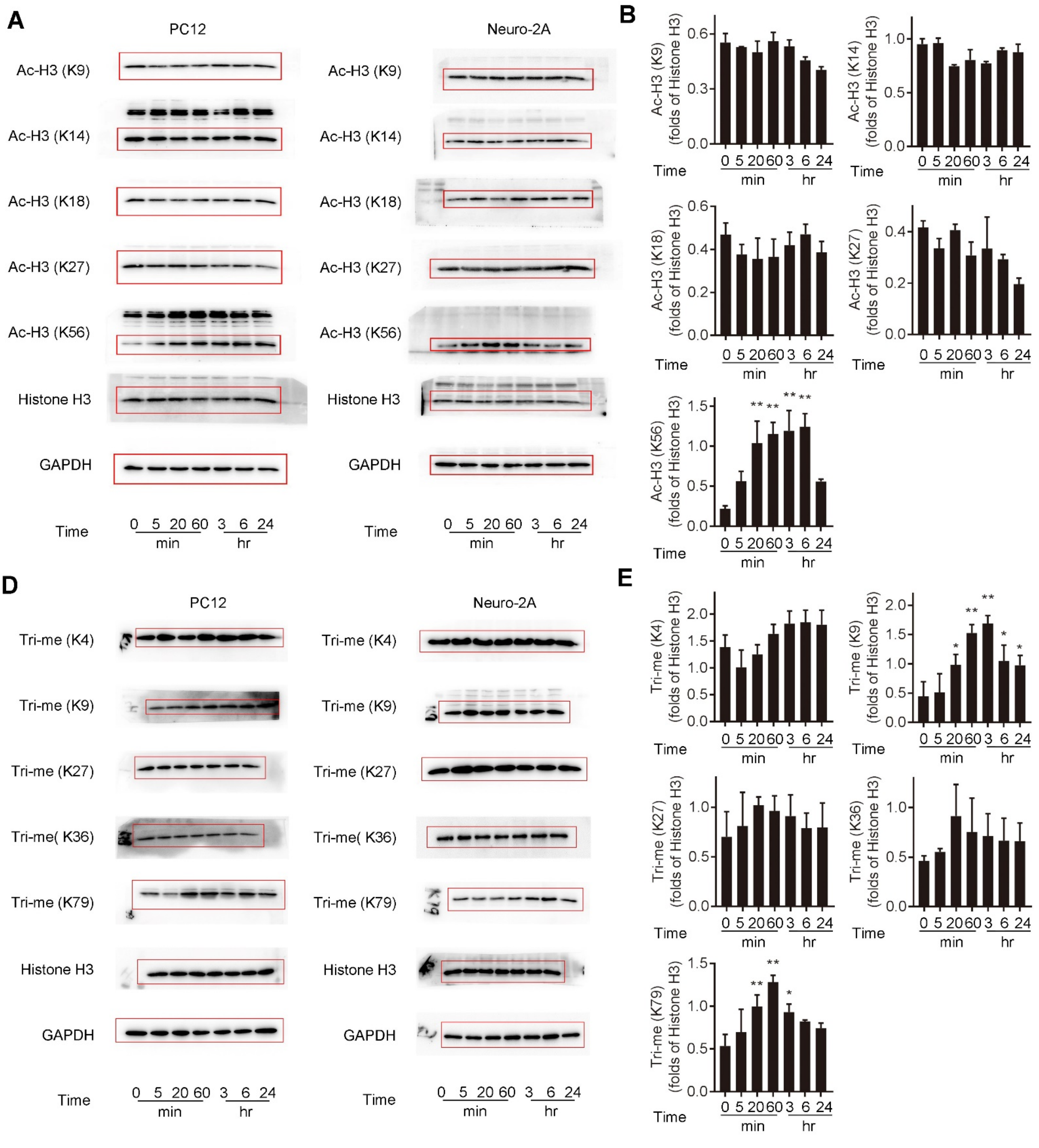

(A and B) Full western blots and quantification analysis. PTA increased acetyl-histone H3 (Lys56) in Neuro-2A and PC12 cells. Related to Figure 4A. (C and D) Full western blots and quantification analysis. PTA increased trimethyl-histone H3 (Lys9 and 79) in Neuro-2A and PC12 cells. Related to Figure 4B.

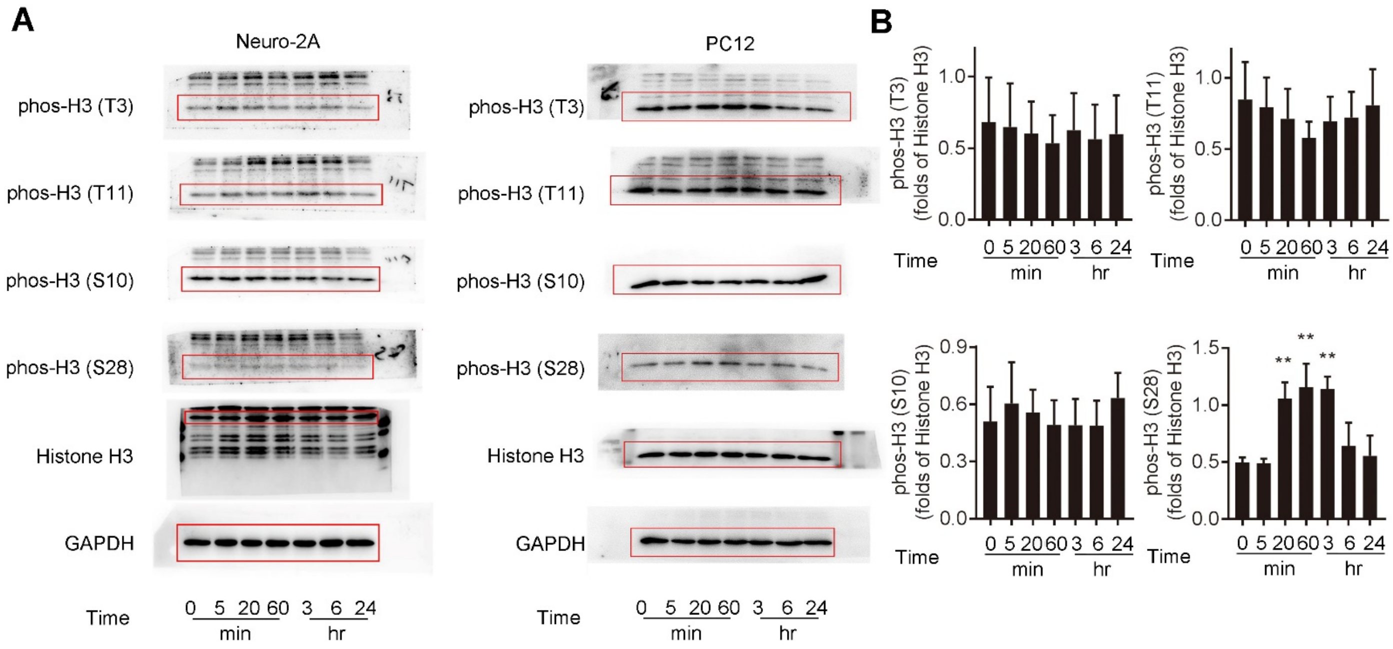

(A and B) Full western blots and quantification analysis. PTA increased phos-histone H3 (Ser28) in Neuro-2A and PC12 cells. Related to Figure 4C.

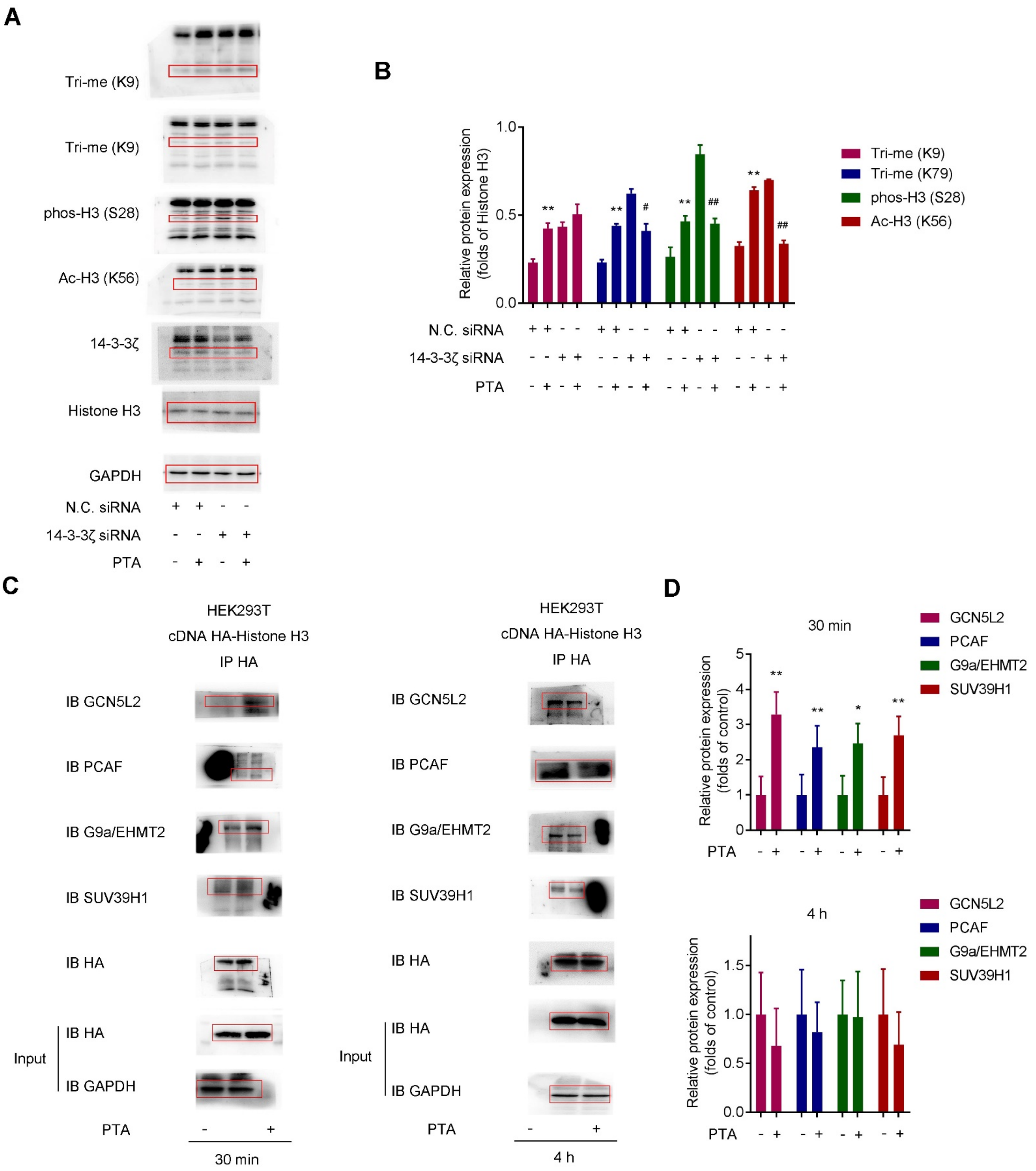

(A and B) Full western blots and quantification analysis. 14-3-3ζ knockdown blocked PTA-dependent post-translational modifications of histone H3. Related to Figure 4E. (C and D) Full western blots and quantification analysis. PTA promoted interactions of histone acetyltransferases (HATs) and histone methyltransferases with histone H3. Related to Figure 4F.

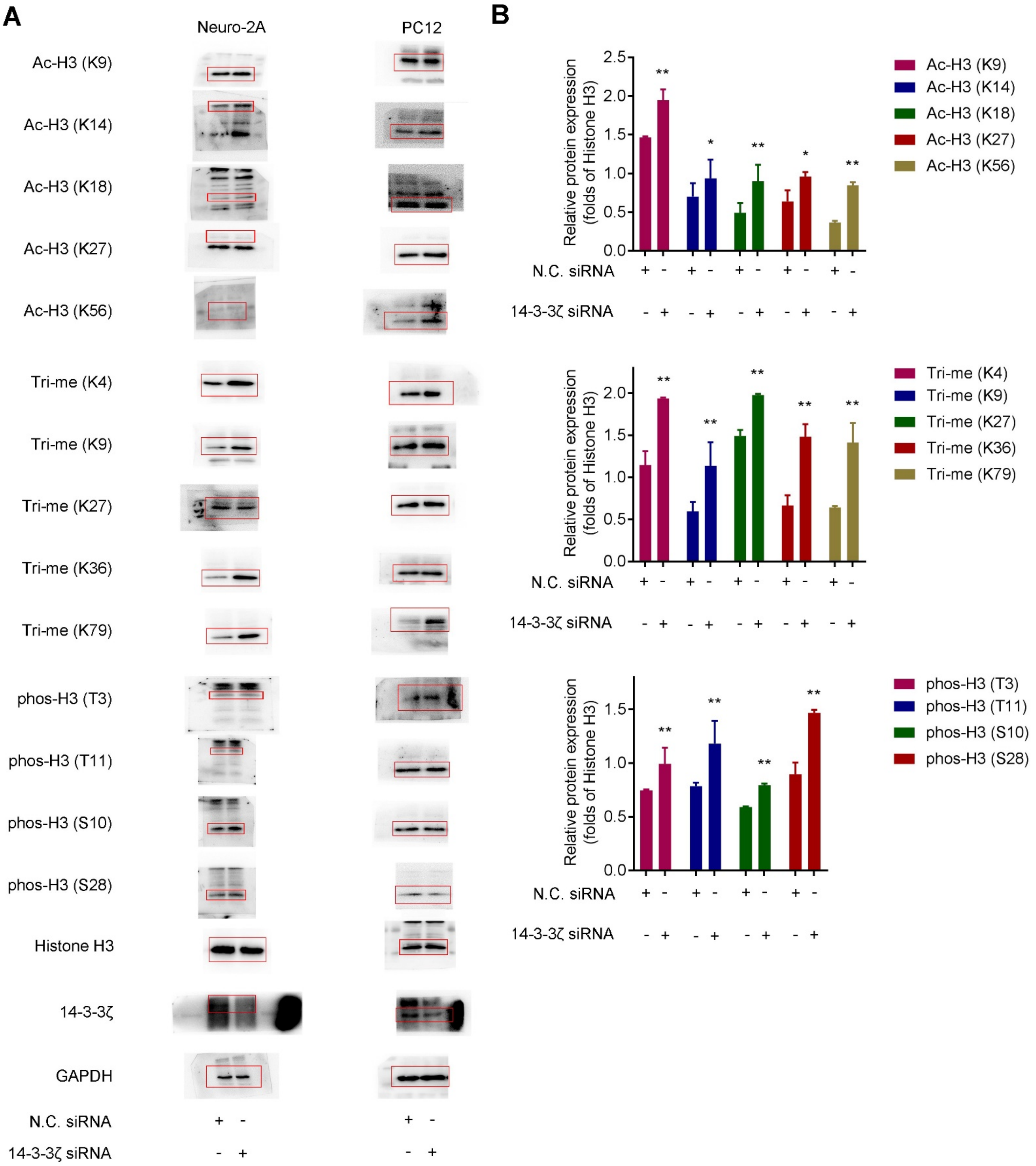

(A and B) Full western blots and quantification analysis. siRNA 14-3-3ζ regulated multiple post-translational modifications on histone H3. Related to Figure S4A.

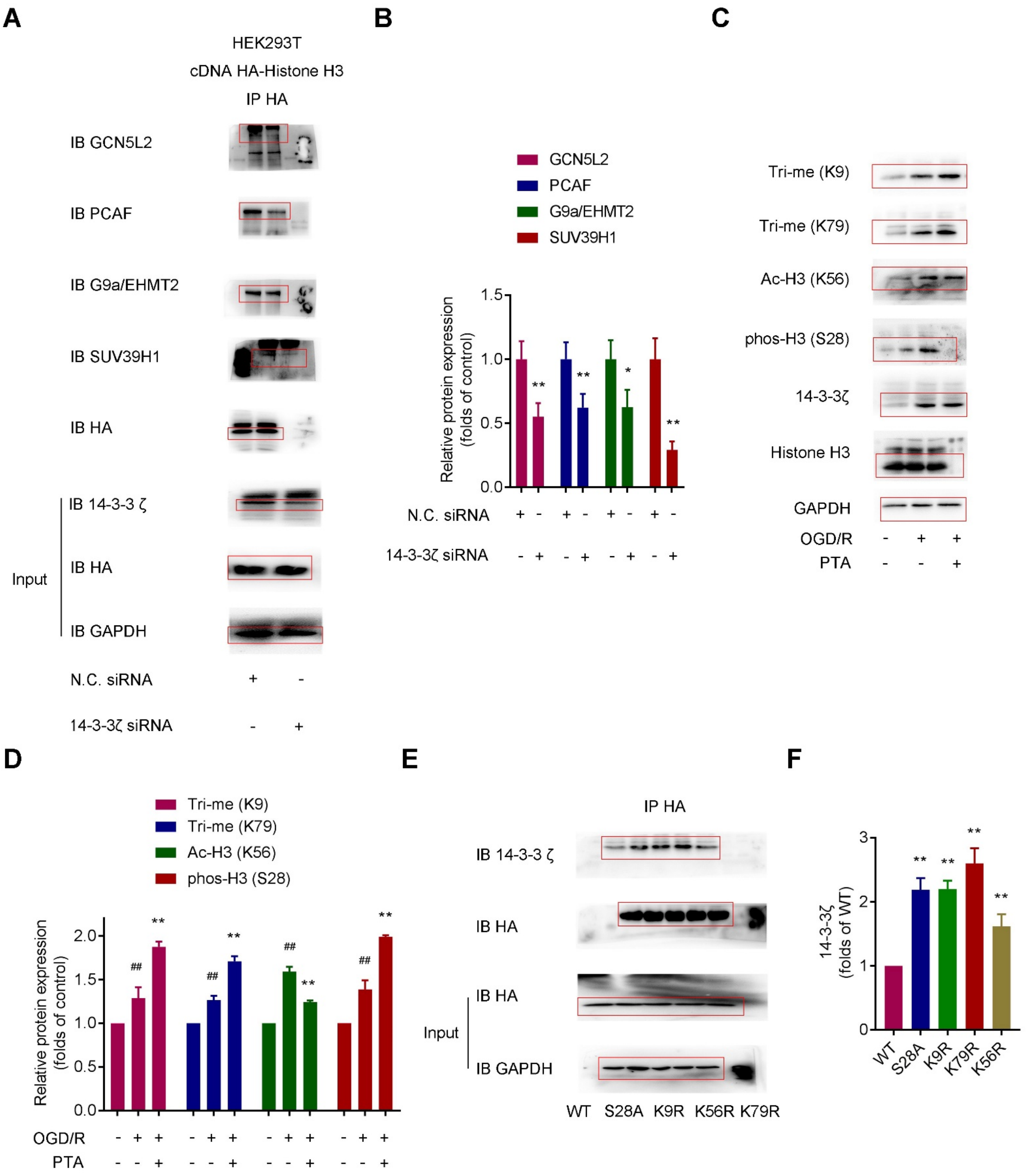

(A and B) Full western blots and quantification analysis. siRNA 14-3-3ζ did not show obvious effect on interaction of histone acetyltransferases (HATs) and histone methyltransferases with histone H3. Related to Figure S4B. (C and D) Full western blots and quantification analysis. PTA could regulate acetylation, trimethylation and phosphorylation levels of 14-3-3ζ in OGD/R model. Related to Figure S4C. (E and F) Full

western blots and quantification analysis. Modification site mutations promoted interaction of 14-3-3ζ with histone H3. Related to Figure S4D.

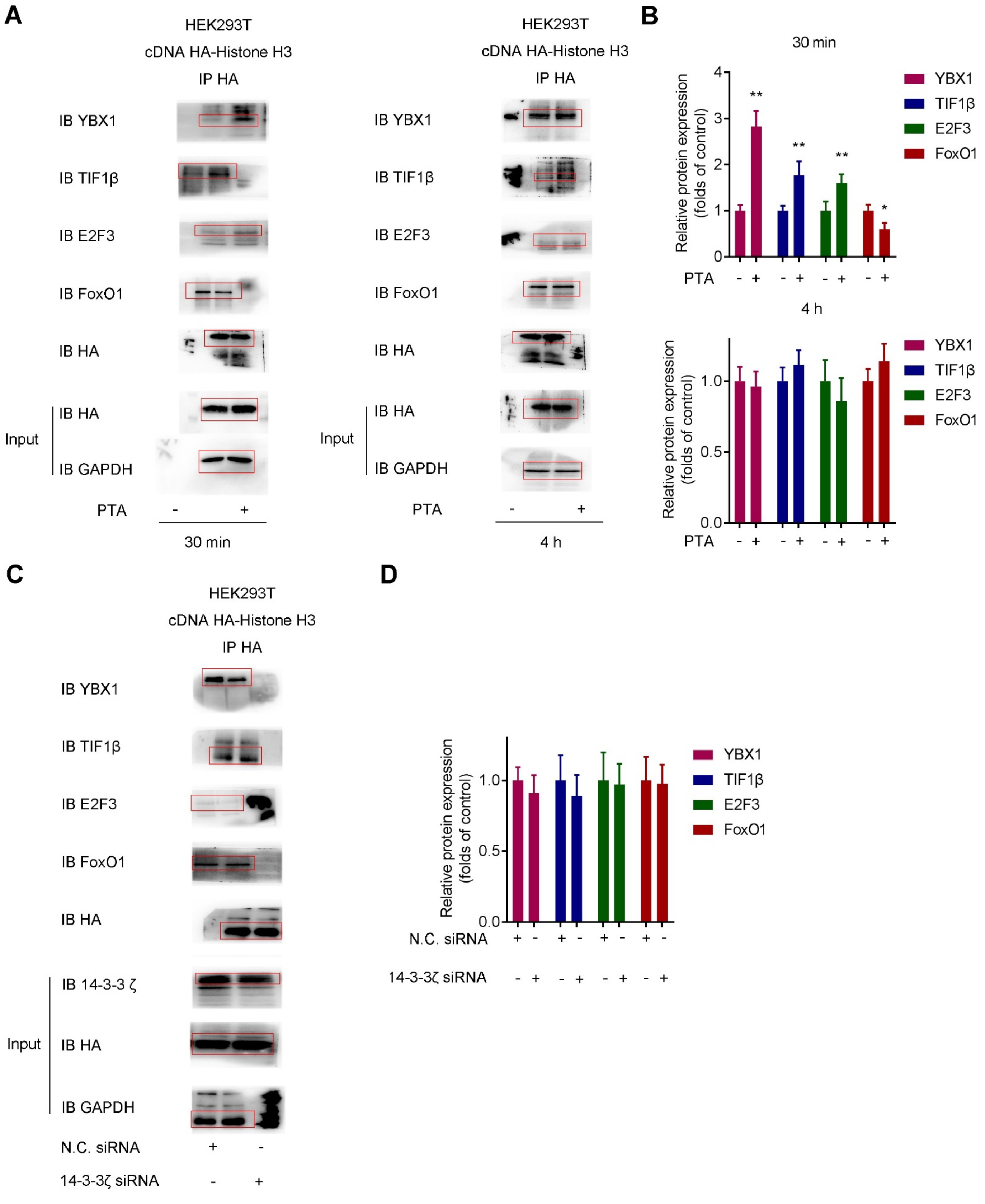

(A and B) Full western blots and quantification analysis. Histone H3 interaction with transcription factors was promoted by PTA in HA-tagged histone H3-transfected HEK293T cells. Related to Figure 5J. (C and D) Full western blots and quantification analysis. 14-3-3ζ knockdown did not affect histone H3 binding to transcription factors. Related to Figure 5K.

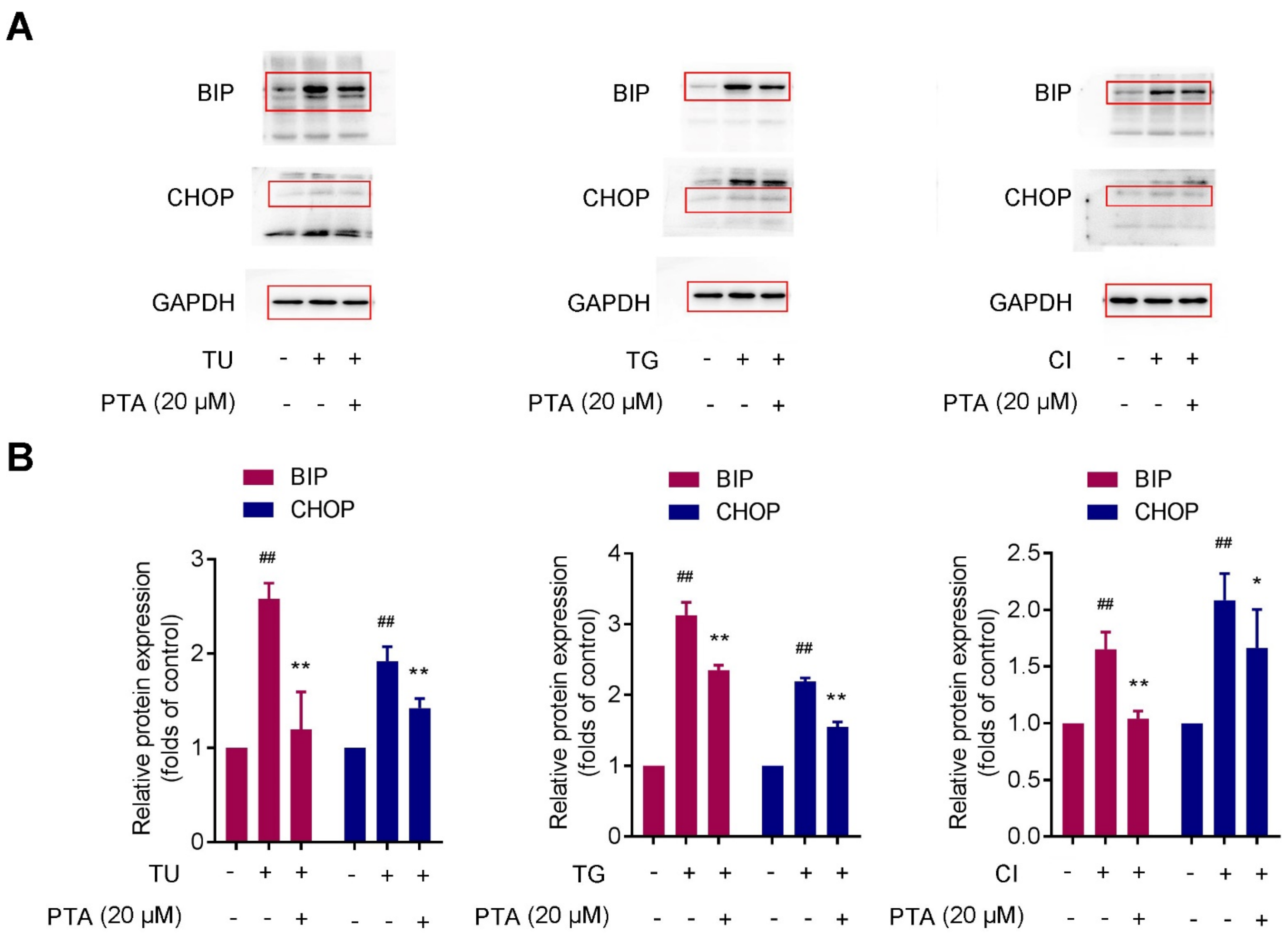

(A and B) Full western blots and quantification analysis. PTA inhibited expressions of ER stress-related proteins BIP and CHOP in Neuro-2A cells. Related to Figure 7E.
